# Sex and Genetic Background Influence Superoxide Dismutase (cSOD)-Related Phenotypic Variation in *Drosophila melanogaster*

**DOI:** 10.1534/g3.117.043836

**Published:** 2017-06-17

**Authors:** Courtney E. Lessel, Tony L. Parkes, Joel Dickinson, Thomas J. S. Merritt

**Affiliations:** * Department of Chemistry and Biochemistry, Laurentian University, Sudbury, Ontario P3E 2C6, Canada; † Biology and Chemistry Department, Nipissing University, North Bay, Ontario P1B 8L7, Canada; ‡ Department of Psychology, Laurentian University, Sudbury, Ontario P3E 2C6, Canada

**Keywords:** superoxide dismutase, *Drosophila melanogaster*, sex, genetic background

## Abstract

Mutations often have drastically different effects in different genetic backgrounds; understanding a gene’s biological function then requires an understanding of its interaction with genetic diversity. The antioxidant enzyme cytosolic copper/zinc superoxide dismutase (cSOD) catalyzes the dismutation of the superoxide radical, a molecule that can induce oxidative stress if its concentration exceeds cellular control. Accordingly, *Drosophila melanogaster* lacking functional cSOD exhibit a suite of phenotypes including decreased longevity, hypersensitivity to oxidative stress, impaired locomotion, and reduced NADP(H) enzyme activity in males. To date, cSOD-null phenotypes have primarily been characterized using males carrying one allele, *cSod^n108^red*, in a single genetic background. We used ANOVA, and the effect size partial eta squared, to partition the amount of variation attributable to cSOD activity, sex, and genetic background across a series of life history, locomotor, and biochemical phenotypes associated with the cSOD-null condition. Overall, the results demonstrate that the cSOD-null syndrome is largely consistent across sex and genetic background, but also significantly influenced by both. The sex-specific effects are particularly striking and our results support the idea that phenotypes cannot be considered to be fully defined if they are examined in limited genetic contexts.

Biological phenotypes are not single gene phenomena, but instead are functions of complex interactions between multiple loci, the environment, and chance. Further, phenotypes, even if governed by single major effect loci, are generally sensitive to genetic background, *i.e.*, molecular variation across the genome (Ayroles *et al.* 2009; Chandler *et al.* 2013; Chari and Dworkin 2013). Moreover, different genetic backgrounds vary in their ability to buffer genetic and environmental perturbations, further contributing to complex trait variation (Chandler *et al.* 2013; Chari and Dworkin 2013). Loci that act as modifiers of complex traits do so through interaction with variation across the rest of the genome; consequently, more complex biological networks, involving numerous genes, are likely more susceptible to genetic background effects than simpler networks (Spencer *et al.* 2003; Chandler *et al.* 2013; Chari and Dworkin 2013; Bing *et al.* 2014).

Many complex traits also exhibit sexual dimorphism, or sex-specific variation in phenotypes, including differences in morphology, physiology, biochemistry, behavior, or life history strategy (Ranz *et al.* 2003; Fairbairn and Roff 2006; Jordan *et al.* 2007; Yamamoto *et al.* 2009; Assis *et al.* 2012). Moreover, sexual dimorphism is a component of sex determination, the hierarchy of events resulting in the development of sexual characteristics (Clough and Oliver 2012). In *Drosophila melanogaster*, sex determination is controlled by a gene cascade and differential regulation of loci during sex differentiation could contribute to genetic background-induced variation (Clough *et al.* 2014).

Understanding the translation of genotype to phenotype relies on understanding the complex interactions that occur within and between loci. This understanding is limited and compromised when studies use single, isogenic backgrounds or single sexes (Phillips *et al.* 1989; Radyuk *et al.* 2004; Bernard *et al.* 2011). That said, it can be difficult to accurately define and quantify appropriate phenotypes by which to assess genetic background and sex effects. Current understanding of genetic interactions is largely based on the examination of the effects of mutant alleles on phenotypes [*e.g.*, SOD; Parkes *et al.* (1998a,b) and Bernard *et al.* (2011)], in controlled genetic backgrounds and environments. This approach simplifies the way mutational effects are analyzed, but also potentially biases how allelic effects are interpreted [reviewed in Chandler *et al.* (2013)]. Instead, it is important to realize that genetic background variation can contribute to phenotypic variation, even when examining the influence of a single mutant allele [*e.g.*, Rzezniczak and Merritt (2012) and Bing *et al.* (2014)].

In most organisms, SOD, as a component of the antioxidant enzyme defense network (Phillips *et al.* 1989; Michiels *et al.* 1994), is the primary scavenger of the reactive oxygen species (ROS) superoxide (O_2_^−^; McCord and Fridovich 1969). ROS are a biologically important class of molecules produced both as metabolic by-products and in a coordinated manner for essential metabolic regulation (Ozcan and Ogun 2015), and balancing ROS is critical to cell function. In ROS metabolism, SOD converts superoxide to hydrogen peroxide (H_2_O_2_), which catalase and a variety of peroxidases further reduce to water (Phillips *et al.* 1989; Michiels *et al.* 1994). As the superoxide anion is largely membrane impermeable, it primarily produces local effects (Bafana *et al.* 2011). Several SOD isoforms also localized in the cell therefore exist, differing in catalytic mechanisms, metal ion cores, and physiological function, each of which scavenge superoxide in distinct cellular compartments (Bafana *et al.* 2011). cSOD, the most abundant eukaryotic SOD isoform, is located in the cytosol, mitochondrial intermembrane space, lysosomes, and the nucleus (Zelko *et al.* 2002; Bafana *et al.* 2011). A second SOD isoform, mitochondrial (manganese) SOD (MnSOD), is localized to the mitochondrial matrix, and functions independently of cSOD (Duttaroy *et al.* 1997; Zelko *et al.* 2002).

*D. melanogaster* lacking cSOD activity (cSOD-null homozygotes) are unable to metabolize superoxide, and therefore exist in a state of chronic oxidative stress (Bernard *et al.* 2011), resulting in a set of pathological conditions collectively known as the cSOD-null syndrome (Phillips *et al.* 1989; Parkes *et al.* 1998a). This syndrome is characterized by a suite of phenotypes ranging from behavioral and life history differences [*e.g.*, changes in locomotion, Martin *et al.* (2009) and Jones and Grotewiel (2011); viability, Sun and Tower (1999); and longevity, Phillips *et al.* (1989), and Parkes *et al.* (1998b)], to biochemical differences [*e.g.*, metabolomic, Knee *et al.* (2013) and NADP(H) enzyme activity, Bernard *et al.* (2011)]. Although cSOD function is often studied by quantifying phenotypic changes, predominantly in males with isogenic backgrounds (Parkes *et al.* 1998a; Bernard *et al.* 2011; Knee *et al.* 2013), the syndrome phenotypes suggest that the system is better studied as a complex trait. Viability and longevity, life history phenotypes, and negative geotaxis, a locomotor phenotype, are depressed in cSOD-null flies (Phillips *et al.* 1989; Parkes *et al.* 1998b; Sun and Tower 1999; Martin *et al.* 2009). Similarly, the NADP(H) enzymes, Malic enzyme (MEN), Isocitrate dehydrogenase (IDH), and Glucose-6-phosphate dehydrogenase (G6PD), have reduced activity in cSOD-null male flies (Bernard *et al.* 2011). These enzymes reduce NADP+ to NADP(H), a key cofactor used by catalase and glutathione-dependent antioxidants in scavenging hydrogen peroxide produced by SOD (Kanzok *et al.* 2001). Furthermore, many cSOD phenotypes, including longevity (Spencer *et al.* 2003), locomotion (Jordan *et al.* 2007; Yamamoto *et al.* 2009), and biochemistry (Ranz *et al.* 2003; Merritt *et al.* 2009), are known to have a sex-specific component. Acknowledging the limitations of examining interactions within relatively simple genetic systems has led to a realization of the need to improve our understanding of how genes function in complex, biologically relevant, genetic systems.

In this paper, we examine the influence of sex and genetic background on a set of cSOD-associated life history, locomotor, and biochemical phenotypes across a range of cSOD activities: 0–80% of wild-type (WT). We hypothesized that differences in the ROS state across cSOD activities would manifest as variation in response to sex and genetic background, with different genetic backgrounds potentially ameliorating or enhancing the phenotypic effects associated with the cSOD-null syndrome. Our results show that large-scale differences in cSOD activity result in pervasive phenotypic changes, and that these changes are significantly modified, both enhanced or suppressed, by differences in sex and genetic background. Higher levels of cSOD activity generally resulted in phenotypes closer to WT, which were more susceptible to modification by sex and genetic background.

## Materials and Methods

### Fly stocks and lines

Second chromosome substitution lines were generated using marker-assisted introgression and a strategically selected subset of eight isofemale lines from the *D. melanogaster* Genetic Reference Panel (DGRP) lines established by the Mackay Lab (Mackay *et al.* 2012): 304, 307, 313, 324, 335, 517, 705, and 820. The subset was chosen, using available DGRP phenotype data (Mackay *et al.* 2012), to include one high- and one low-magnitude line each for four cSOD-related phenotypes: longevity, fitness, paraquat sensitivity, and startle response. We placed these eight DGRP 2nd chromosomes into a common genetic background: *w^1118^*; *DGRPi/CyO*; *VT83*, with “*i*” being the 2nd chromosome from one of the DGRP lines and *VT83* being a 3rd chromosome isolated from the wild (Merritt *et al.* 2006).

In *D. melanogaster*, the cSOD locus is on the 3rd chromosome, and the active cSOD enzyme is a homodimer (Campbell *et al.* 1986). We used a matched set of cSOD-null and WT cSOD alleles, previously described by Campbell *et al.* (1986), Phillips *et al.* (1989), and Parkes *et al.* (1998a,b). Briefly, the cSOD-null line (*T0*) possesses the *cSod^n108^red* null allele on the 3rd chromosome (Parkes *et al.* 1998a,b), while the parallel control line (*T5*) possesses this same null allele on the 3rd chromosome in conjunction with a *cSod* transgene on the 2nd chromosome, under control of the native *cSod* promoter, creating a whole organism transgenic rescue line (Parkes *et al.* 1998a,b). The *T*0 and *T*5 lines differ only in the absence or presence of the transgene. When homozygous, the transgene restores ∼70% of WT cSOD activity (Parkes *et al.* 1998a,b) and *T5* flies have been phenotypically indistinguishable from WT cSOD flies in previous studies (Parkes *et al.* 1998b; Bernard *et al.* 2011).

**Figure 1:**
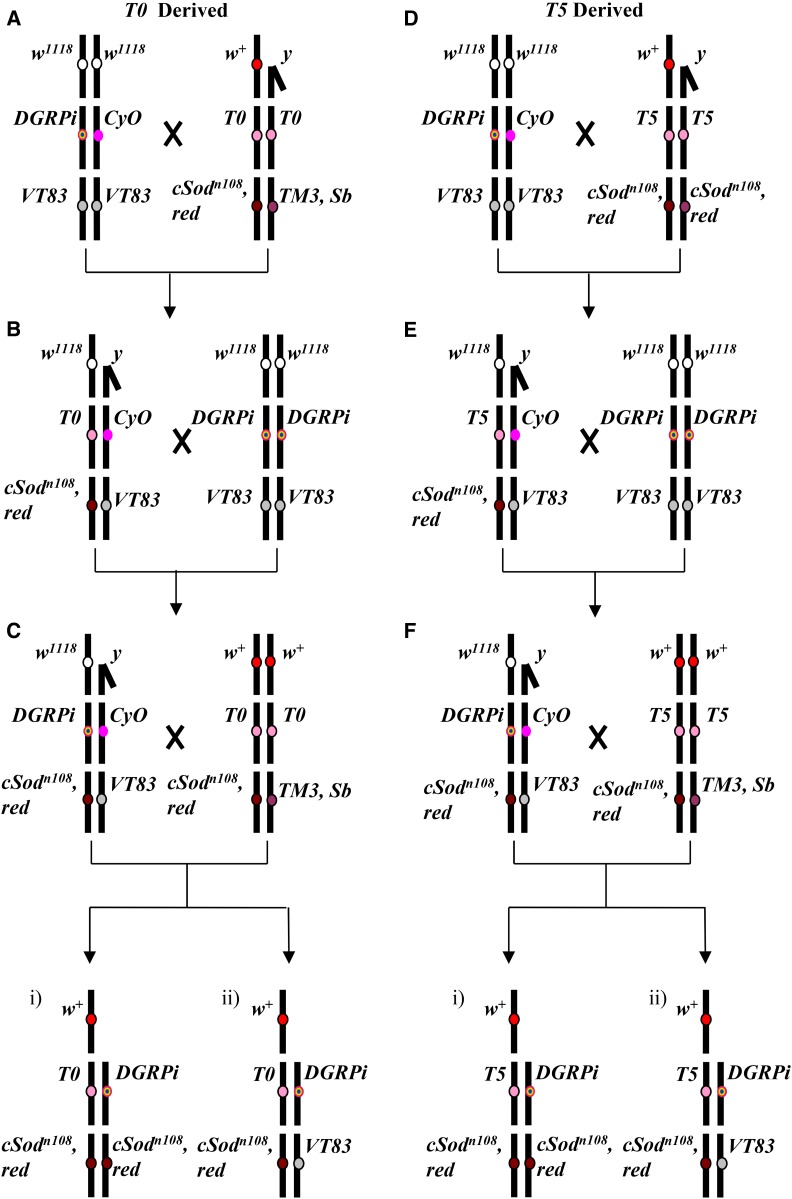
Crossing schemes used to generate 0 and 50% cSOD flies (A–C) and 30 and 80% cSOD flies (D–F). In all crosses adults were placed on fresh cornmeal-corn syrup-yeast-agar media and allowed to mate and lay eggs for 1 wk, moved to fresh media for 1 wk, then discarded. Crosses (A, D) and (B, E) were set up using two male and two virgin female flies. Single males were crossed to two virgin females in cross (C, F) as male progeny from cross (B, E) expressing the curly wing phenotype could have possessed one of two genotypes, only one of which was desired for cross (C, F). Use of single males prevented mixing males with different genotypes and allowed crosses that had been set up with the incorrect genotype to be discarded. Cross (C) generated (i) *w^+^;T0/DGRP^i^;csod^n108^,red/csod^n108^,red* (0% wild-type cSOD activity) and (ii) *w^+^*; *T0/DGRPi;csod^n108^,red/VT83* (50% wild-type cSOD activity). Cross (C) also produces *w^+^;T0/CyO;csod^n108^,red/csod^n108^,red*, *w^+^;T0/CyO*; *csod^n108^,red/VT83*, *w^+^;T0/CyO*; *csod^n108^,red/TM3*, *ser*, *w^+^;T0/CyO*; *VT83/TM3*, *ser*, *w^+^;T0/DGRP^i^*; *csod^n108^,red/TM3*, *ser*, and *w^+^;T0/DGRP^i^*; *VT83/TM3*, *ser* genotypes. Cross (F) generated (i) *w^+^;T5/DGRPi;csod^n108^,red/csod^n108^,red* (30% wild-type cSOD activity) and (ii) *w^+^*; *T5/DGRPi;csod^n108^,red/VT83* (80% wild-type cSOD activity). Cross (F) also produces *w^+^;T5/CyO;csod^n108^,red/csod^n108^,red*, and *w^+^;T5/CyO*; *csod^n108^,red/VT83* progeny. Across genotypes, female flies were heterozygous at the X chromosome for *w^+^/w^1118^*, while males were hemizygous for the *w^+^* allele. The four genotypes carrying the *TM3*, *Sb* 3rd chromosome balancer, produced by (C), are distinguished by a stubble bristles phenotype and have been removed from analysis as the four genotypes encompass two phenotypes that are visually indistinguishable and possess different levels of cSOD activity. The genotypes carrying the *CyO* chromosome, produced in (C) and (F), have been excluded from the analyses as preliminary results suggested the balancer chromosome was falsely driving genetic background differences. cSOD, cytosolic copper/zinc superoxide dismutase.

To quantify the effects of cSOD activity, sex, and genetic background on phenotypes, we crossed the *T0* and *T5* lines to the background-replaced iso 2nd chromosome lines to generate two cSOD activity ranges: a low range (0 and 50% of WT activity flies) and a middle range (30 and 80% of WT activity flies). Distinct schemes were designed to extract both 2nd and 3rd chromosomes from the *T0* (low cSOD activity; Figure 1, A–C) and *T5* (moderate cSOD activity; Figure 1, D–F) lines because elements contributing to cSOD activity are located across both chromosomes (2nd for the transgene and 3rd for the native locus). The Scheme 1 crosses created progeny with either 0 or 50% of WT cSOD activity (*w^+^*; *T0/DGRPi*; *csod^n108^,red/ csod^n108^,red* and *w^+^*; *T0/ DGRPi*; *csod^n108^,red/VT83*, respectively; Figure 1, Ci and Cii), while the Scheme 2 crosses created progeny with either 30 or 80% of WT cSOD activity (*w^+^*; *T5/DGRPi*; *csod^n108^,red /csod^n108^,red* and *w^+^*; *T5/DGRPi*; *csod^n108^,red/VT83*, respectively; Figure 1, Fi and Fii). For each phenotype assay, we generated progeny of the appropriate genotypes via five replicate crosses of each crossing scheme and genetic background (two schemes, eight backgrounds, and five replicates = 80 separate crosses). All experimental flies were assumed to be nonvirgin as they were collected 48 hr post eclosion. Strains are available upon request.

### Genotypic viability assay

Genotypic viability was assessed as described by Merritt *et al.* (2006) using flies generated from the crosses shown in Figure 1, C and F). Adult flies from each cross were counted for 1 wk (7 d) from first eclosion, and the frequencies of occurrence of each genotype were calculated out of the total number of flies collected for each cross. Due to the variation in success across lines, the total number of animals assayed varied between 2660 flies and 1928 flies, for the 0 and 50% comparison and the 30 and 80% comparison, respectively.

### Longevity assay

Longevity, based on genotype mortality, was measured as described by Parkes *et al.* (1998b). Adult flies of each genotype were collected 48 hr post eclosion and transferred to standard 25 × 95 mm shell vials with a maximum of 20 flies per vial. Longevity was measured in single-sex, single-genotype, vials—maintained at 25°, 12 hr light: dark cycle—and mortality was recorded every 2 d. Flies were transferred to fresh media every 4 d until no living flies remained. Due to the variation in success across lines, the total number of animals assayed varied between 2260 flies and 1705 flies, for the 0 and 50% comparison and the 30 and 80% comparison, respectively.

### Negative geotaxis assay

Adult negative geotaxis (hereafter simply “geotaxis”) was measured using an assay modified from Patel and Tamanoi (2006) and Sofola *et al.* (2010). Briefly, five replicates of 15 flies of each genotype were collected 48 hr post eclosion, and aged for 3 d in single-sex, single-genotype, vials. Following aging, the groups of flies were transferred into empty 25 × 95 mm shell vials marked with a height of 5 cm and allowed 30 sec to recover. Following recovery, flies were tapped down to the bottom of the vial and allowed 10 sec to climb. After 10 sec, the number of flies above and below the 5 cm mark was counted by eye. For each vial, three tap down trials were performed, each at 1 min intervals. A performance index (PI), an estimate of the probability that a fly will climb, was calculated for each genotype following Sofola *et al.* (2010). PI values range from zero to one; values close to one indicate that flies have a high geotaxic response, while values close to zero indicate that flies have a poor geotaxic response (Sofola *et al.* 2010). PI was calculated as PI = 1/2 (*n*_TOTAL_+ *n*_TOP_ − *n*_BOTTOM_ / *n*_TOTAL_), where *n*_TOTAL_ is the total number of flies, *n*_TOP_ is the number of flies above the 5 cm line, and *n*_BOTTOM_ is the number of flies below the 5 cm line (Sofola *et al.* 2010). Due to the variation in success across lines, the total number of animals assayed varied between 2168 flies and 1923 flies, for the 0 and 50% comparison and the 30 and 80% comparison, respectively.

Because cSOD-null flies are strikingly poor climbers, we modified the geotaxis assay by using a shorter climbing distance than typically employed (Patel and Tamanoi 2006; Sofola *et al.* 2010). However, this modification could reduce the ability of the assay to detect small geotaxic differences in high-performance flies (*i.e.*, animals possessing 30, 50, and 80% cSOD activity). Such a reduction in resolving power could lead us to detect no difference across backgrounds, even if small differences exist. Therefore, we conducted a second locomotor assay, the countercurrent climbing assay (Petersen *et al.* 2013), to more closely assess potential performance differences across genotypes.

### Countercurrent climbing assay

Adult climbing ability was measured using a countercurrent climbing assay (hereafter simply “countercurrent”) as described by Petersen *et al.* (2013). The countercurrent apparatus consists of two sets of four shell vials taped together, with the bottom set labeled 1–4 and the top set labeled 5–8 (Supplemental Material, Figure S1 in File S1; Petersen *et al.* 2013). Five replicates of ∼15 adult flies of each genotype and sex were collected 48 hr post eclosion, combined, and aged for 3 d in standard 25 × 95 mm shell vials in single-sex, mixed-genotype, vials. For each test vial, the number of flies from each genotype was counted, out of the mixed genotype group, post assay (Benzer 1967). Aged flies were loaded into vial 1 and vial 8 was inverted over vial 1 (Figure S1B in File S1), the vials were tapped down, and the flies given 1 min to climb. Following 1 min, the top set of vials was shifted over, the vials tapped down, and the flies were allowed 1 min to climb (Figure S1B in File S1). This process was repeated a total of seven times, with vials unopposed by other vials plugged to prevent flies from escaping (Figure S1B in File S1). Flies were classified by climbing ability based on the vials they were in at the end of the trial: poor = vial 1, moderate = vials 2–4, or good = vials 5–8 (Figure S1B in File S1; Petersen *et al.* 2013). A partition coefficient (CF), an estimate of the probability that a fly will climb out of its starting vial at each trial, was calculated for each genotype (Kamikouchi *et al.* 2009). CF values range from zero to one; values close to one indicate that flies tend to climb up, while values close to zero indicate that flies tend to not climb (Kamikouchi *et al.* 2009). CF was calculated as *CF* = Ʃ*Nk*(*k*−1)/(*n*−1)Ʃ*Nk*, where *n* = the number of climbing classes, *Nk* is the number of flies in the *k*th climbing class, and the climbing classes were assigned the *k* values poor (*k* = 1), moderate (*k* = 2), and good (*k* = 3; Kamikouchi *et al.* 2009). Analyses were rerun using the vial that each fly ended up in as a class (eight potential classes) and the results were consistent with the binned analyses (discussed below). Therefore, we only show the data and results from the binned analyses. Due to the variation in success across lines, the total number of animals assayed varied between 2106 flies and 1962 flies, for the 0 and 50% comparison and the 30 and 80% comparison, respectively.

### Enzyme activity assays

For all enzyme assays, adult flies were collected 48 hr post eclosion, aged for 3 d, and frozen at −80° in single-sex, single-genotype groups of four flies. Prior to homogenization, samples were weighed to the nearest 0.01 mg with a microbalance (MX5 Balance, Mettler Toledo AG, Greifensee, Switzerland). All enzyme reactions were performed in a standard 96-well microtiter plate and absorbance measured with a microplate spectrophotometer (SpectraMax Plus 384, Molecular Devices, Sunnyvale, CA).

#### MEN, IDH, and G6PD enzyme activity assays:

NADP(H) enzyme assays were performed as described by Merritt *et al.* (2006) and Bernard *et al.* (2011). Briefly, fly samples were homogenized in 100 μl of homogenizing buffer per fly (0.1 M TRIS-HCl pH 7.4 and 0.01 M NADP^+^), centrifuged at 13,000 rpm for 12 min at 4°, and the supernatant collected. Within each microplate well, the reaction mixture consisted of 10 μl of sample supernatant and 100 μl of the assay solution (MEN: 0.1 M TRIS-HCl pH 7.4, 10 mM malate, 5 mM MnCl_2_, and 0.34 mM NADP+; IDH: 0.1 M TRIS-HCl pH 8.6, 1.37 mM isocitrate, 0.84 mM MgSO_4_, and 0.1 mM NADP+; and G6PD: 20 mM TRIS-HCl pH 7.4, 3.5 mM G6P, and 0.2 mM NADP+). NADP(H) produced was quantified as an increase in absorbance measured at 340 nm. For the MEN and IDH reactions, absorbance was measured at 25° every 9 sec for 3 min. For the G6PD reaction, absorbance was measured at 25° every 9 sec for 5 min. Enzyme activities for each genotype sample were calculated from the mean enzyme activity of three technical replicates. The total number of animals assayed was 1184 flies and 1248 flies, for the 0 and 50% comparison and the 30 and 80% comparison, respectively. Genes for MEN and IDH in *D. melanogaster* are located on the 3rd chromosome, while the gene for G6PD is located on the X chromosome. Therefore, differences in the 2nd chromosome genetic background will not change the MEN, IDH, and G6PD alleles present in the experimental flies.

#### cSOD enzyme activity assay:

cSOD activity was quantified using a commercial assay kit (Cayman Chemical Superoxide Dismutase Assay Kit, Ann Arbor, MI, Product Number: 706002) following the manufacturer’s suggested protocol with minimal modification. Briefly, fly samples were homogenized in chilled 20 mM HEPES buffer (pH 7.4, 1 mM EDTA, 210 mM mannitol, and 70 mM sucrose) at a ratio of 100 μl/fly and then diluted to a final ratio of 400 μl/fly. Fly homogenates were centrifuged at 4° for 10 min at 3000 rpm and the supernatant collected [samples are centrifuged at lower rpm than the NADP(H) samples to prevent MnSOD protein from pelleting]. Prior to assaying, the supernatant was diluted to a ratio of 1:4 supernatant to assay sample buffer. For each sample, two distinct reactions were performed to determine cSOD activity: one for total SOD activity and one for MnSOD activity. cSOD activity is then calculated as the difference between the two. cSOD activity is calculated this way because the cSOD assay cannot distinguish between the two cSOD isoforms, so an inhibitor is required to determine the activity of a single isoform. For total SOD activity (MnSOD and cSOD), the reaction mixture consisted of 10 μl of the diluted sample supernatant, 200 μl of radical detector, and 20 μl of xanthine oxidase. For MnSOD activity, each reaction consisted of 10 μl of the diluted sample supernatant, 190 μl of radical detector, 20 μl of xanthine oxidase, and 10 μl of 5 mM of sodium cyanide (which inactivates cSOD via destabilizing the Cu/Zn complex). All reactions were incubated at room temperature for 30 min and then endpoint absorbance was measured at 25° and 450 nm with a microplate spectrophotometer. Each sample was assayed twice for each reaction (total SOD activity and MnSOD activity) and sample SOD activities were calculated using the equation generated from the SOD standard (bovine erythrocyte Cu/Zn SOD) curve. The total number of animals assayed was 636 flies and 624 flies, for the 0 and 50% comparison and the 30 and 80% comparison, respectively.

### Total protein concentration

Total protein concentration was quantified with a bicinchoninic acid assay using a commercial kit (Pierce, Thermo Scientific, Rockford, IL, Product Number 23225) with modifications following Rzezniczak and Merritt (2012). Within each microplate well, the reaction consisted of 10 μl of sample supernatant and 100 μl of assay reagent. The microplates were incubated at 37° for 20 min and allowed to cool prior to absorbance readings. Endpoint absorbance was measured at 562 nm and at 25° with a microplate spectrophotometer. Total protein concentrations were calculated by comparison with a standard curve generated using 1200 μg/ml, 800 μg/ml, 400 μg/ml, and 100 μg/ml samples of bovine serum albumen. Each sample was assayed three times and mean sample protein concentrations were used to standardize enzyme activities.

### Statistical analysis

ANOVA was used to partition phenotypic variation between cSOD activity, sex, genetic background, their interactions, and the error variance [y = cSOD + sex + genetic background + cSOD*sex + cSOD*genetic background + sex*genetic background + cSOD*sex*genetic background + Error; Leips and Mackay (2000), and Ayroles *et al.* (2009)]. *Post hoc* comparisons were carried out using Tukey’s HSD test. All analyses were performed using JMP 12 statistical software. Separate comparisons were performed on data from crossing Scheme 1 (0 and 50% cSOD activity) and crossing Scheme 2 (30 and 80% cSOD activity) as they were conducted independently and the genotypes obtained from these crosses differed (due to the presence or absence of the cSOD transgene on the 2nd chromosome in the *T0* and *T5* lines). However, general trends across all four activity levels and the two comparisons were noted.

The effect size measure partial eta squared (η^2^*_p_*) was used to estimate the magnitude of variation attributable to each factor and interaction. η^2^*_p_* measures the proportion of variation attributable to a particular factor, while removing variation that is explained by other predictor variables. η^2^*_p_* was determined using the equation η^2^*_p_* = *SS*_Factor_ / *SS*_Factor_ + *SS*_Error_ (Pierce *et al.* 2004) for each factor, and interaction using the Sum of Squares values produced in the ANOVA output.

### Data availability

All strains are available on request. The authors state that all data necessary for confirming the conclusions presented in the article are represented fully within the article.

## Results

Here, we quantify the effects of genetic background and sex in modifying the effect of cSOD activity on a suite of cSOD-sensitive phenotypes. By examining multiple genetic backgrounds across sexes, we were able to partition the amount of variation attributable to cSOD activity, sex, genetic background, and their interactions, for each phenotype. We used the effect size η^2^*_p_* to quantify the proportion of variation attributable to each factor, and all η^2^*_p_* values and their associated statistics can be found in Table S1, Table S2, Table S3, Table S4, Table S5, Table S6, and Table S7 in File S1. Summary tables of maximum and minimum lines effects, average genotype values, and maximum and minimum line values, are illustrated in Table S8, Table S9, and Table S10 in File S1, respectively.

### cSOD activity is controlled by cSOD genotype, but not by second chromosome dominant modifiers

We generated genotypes with a range of cSOD activity levels, across the same eight 2nd chromosome backgrounds (*DGRPi*), using two separate crossing schemes (Figure 1). The cSOD activity for each genotype was initially estimated according to the overall cSOD genotype; however, other studies using isofemale fly lines have shown that 2nd chromosome genetic background can affect cSOD activity (Graf and Ayala 1986). To quantify potential background variation in cSOD activity resulting from the distinct DGRP 2nd chromosomes, we measured cSOD activity across all genotypes and found no significant influence of genetic background on cSOD activity (Figure S2 and Figure S3 in File S1). Large-scale differences in cSOD activity are thus a function of the overall cSOD genotype with only small, statistically insignificant variation across the genetic backgrounds. Hereafter, we refer to the different genotypes by their predicted (and observed) cSOD activities: 0, 50, 30, and 80% cSOD activity. Interestingly, in the 30–80% comparison there was a significant effect of sex on cSOD activity, with activity significantly lower in males than females, though sexual dimorphism in cSOD expression is not observed at the native cSOD locus (Gnad and Parsch 2006; discussed below).

### Life history phenotypes are most affected by cSOD activity with varying sensitivity to sex and genetic background

**Figure 2:**
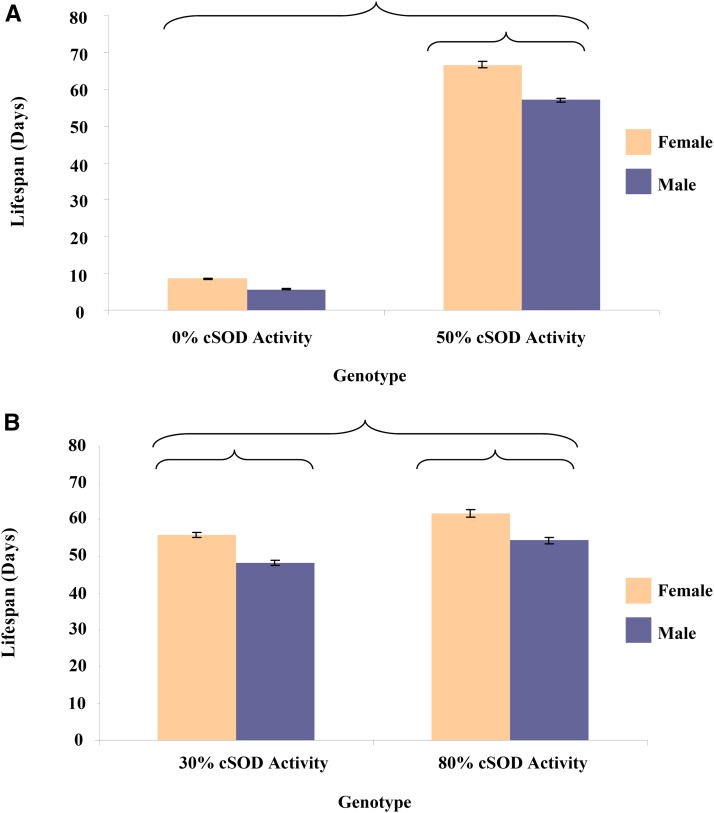
Mean ± SEM longevity (d) for adult male and female flies pooled across the eight *DGRPi* genetic backgrounds within each 3rd chromosome genotype. ANOVA was used to test the factor effects with an α of 0.05. Partial eta squared (η*_p_*^2^) was calculated to quantify the effect of each factor. (A) Sex-by-cSOD activity: *F*_1,2227_ = 35.2, *P* < 0.0001, η*_p_*^2^ = 0.016. (B) cSOD activity: *F*_1,1673_ = 64.9, *P* < 0.0001, η*_p_*^2^ = 0.038; Sex: *F*_1,1673_ = 66.1, *P* < 0.0001, η*_p_*^2^ = 0.038. Brackets enclose significantly different groups. cSOD, cytosolic copper/zinc superoxide dismutase.

**Figure 3:**
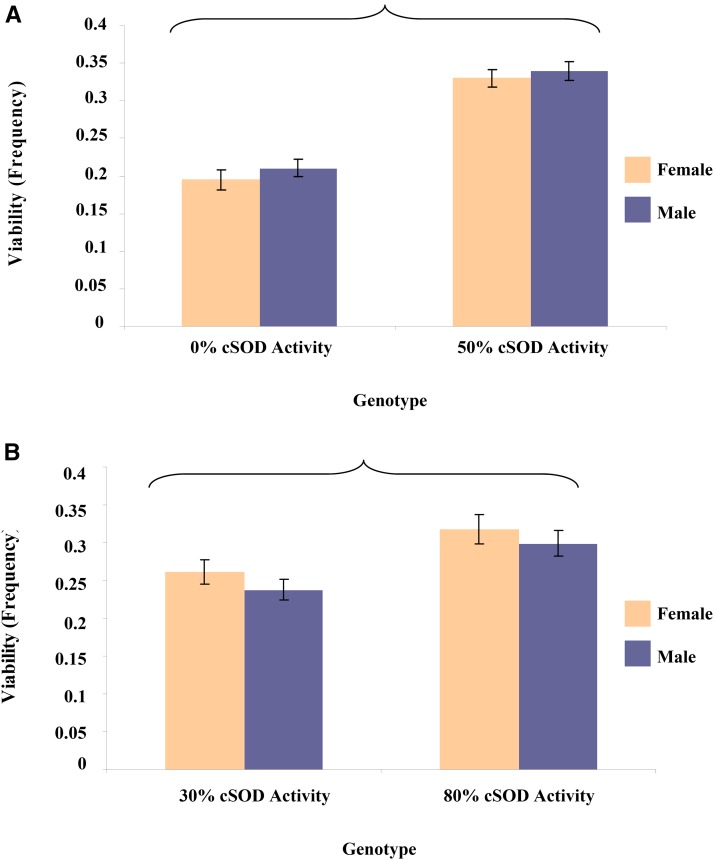
Mean ± SEM of viability (frequency) for adult flies pooled across the eight *DGRPi* genetic backgrounds within each 3rd chromosome genotype. ANOVA was used to test the factor effects with an α of 0.05. Partial eta squared (η*_p_*^2^) was calculated to quantify the effect of each factor. (A) cSOD activity: *F*_1,143_ = 106.7, *P* < 0.0001, η*_p_*^2^ = 0.43. (B) cSOD activity: *F*_1,148_ = 11.2, *P* = 0.001, η*_p_*^2^ = 0.070. Brackets enclose significantly different groups. cSOD, cytosolic copper/zinc superoxide dismutase.

**Figure 4:**
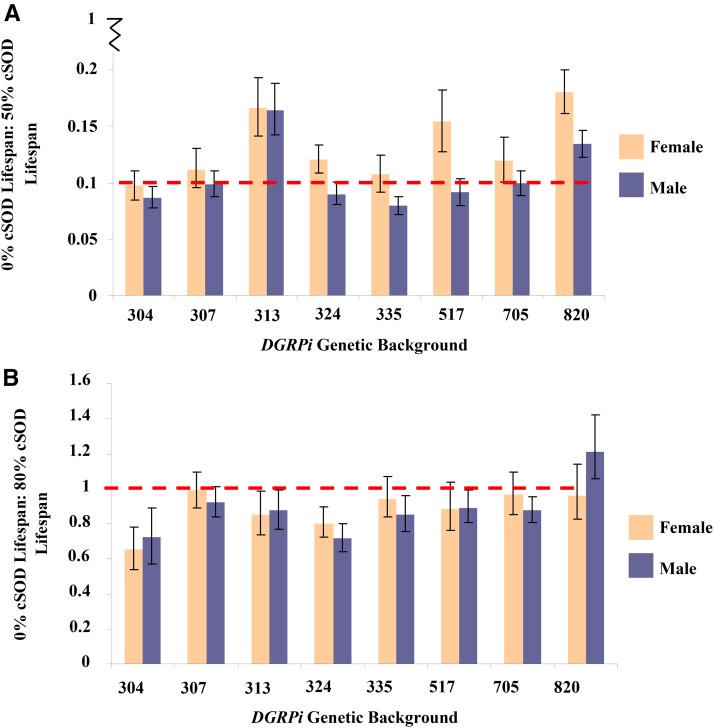
Mean ± SEM of the ratio of low cSOD: high cSOD activity longevities for adult male and female flies across the eight *DGRPi* genetic backgrounds. Values closer to 1 indicate more similar longevities at the levels of cSOD activity being compared, and C.I.s are at 95% and represent the ratio of the low cSOD activity and high cSOD activity SEMs. (A) 0% cSOD activity: 50% cSOD activity: Female: lowest ratio 304, 0.0846 < 0.0972 < 0.111; highest ratio 820, 0.162 < 0.180 < 0.201; Male: lowest ratio 335, 0.0715 < 0.0793 < 0.0873, highest ratio 313, 0.143 < 0.165 < 0.188 (B) 30% cSOD activity: 80% cSOD activity: Female: lowest ratio 304, 0.535 < 0.650 < 0.780; highest ratio 307, 0.889 < 0.990 < 1.10; Male: lowest ratio 324, 0.642 < 0.720 < 0.803; highest ratio 820, 1.05 < 1.21 < 1.42. ANOVA was used to test the factor effects with an α of 0.05. Partial eta squared (η*_p_*^2^) was calculated to quantify the effect of each factor. (A) Sex-by-genetic background-by-cSOD activity, F_7,2227_ = 4.38, *P* < 0.0001, η*_p_*^2^ = 0.0136. (B) Sex-by-genetic background, *F*_7,1673_ = 2.91, *P* = 0.0049, η*_p_*^2^ = 0.0121; genetic background-by-cSOD activity; *F*_7,1673_ = 6.03, *P* < 0.0001, η*_p_*^2^ = 0.0246. cSOD, cytosolic copper/zinc superoxide dismutase.

**Figure 5:**
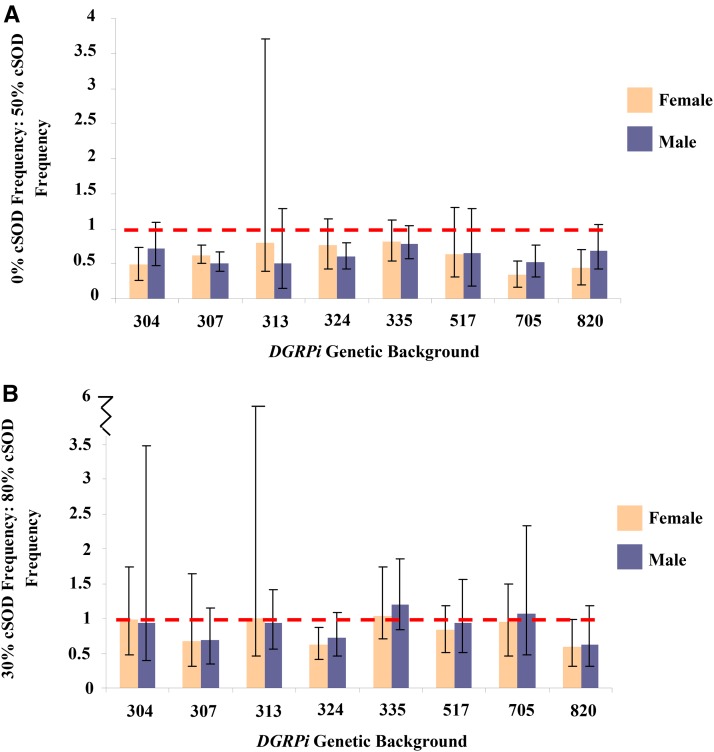
Mean ± SEM of the ratio of low cSOD: high cSOD activity viability (frequency) for adult male and female flies across the eight *DGRPi* genetic backgrounds. Values closer to 1 indicate more similar viabilities at the levels of cSOD activity being compared; C.I.s are at 95% and represent the ratio of the low cSOD activity and high cSOD activity SEMs. (A) 0% cSOD activity: 50% cSOD activity: Female: lowest ratio 705, 0.168 < 0.344 < 0.532; highest ratio 335, 0.538 < 0.819 < 1.12: Male: lowest ratio 313, 0.147 < 0.498 < 1.28; highest ratio 335, 0.575 < 0.781 < 1.05 (B) 30% cSOD activity: 80% cSOD activity, Female: lowest ratio 820, 0.309 < 0.586 < 0.979; highest ratio 335, 0.699 < 1.04 < 1.74: Male: lowest ratio 304, 0.0162 < 0.492 < 0.974; highest ratio 335, 0.720 < 0.902 < 1.08. ANOVA was used to test the factor effects with an α of 0.05. Partial eta squared (η*_p_*^2^) was calculated to quantify the effect of each factor. (A) cSOD activity, *F*_1,143_ = 106.7, *P* < 0.0001, η*_p_*^2^ = 0.427. (B) cSOD activity, *F*_1,148_ = 11.2, *P* = 0.001, η*_p_*^2^ = 0.0704. cSOD, cytosolic copper/zinc superoxide dismutase.

As expected, scores of the life history phenotypes were lowest at 0% cSOD activity and higher at moderate cSOD activity (Figure 2 and Figure 3). Scores were similar between the 50 and 80% cSOD activity groups, suggesting that only 50% cSOD activity was required to recover phenotypes to WT levels. cSOD activity had a larger effect than sex or genetic background on both viability and longevity in the 0–50% comparison. In fact, viability was only affected by cSOD activity, while longevity was also sensitive to sex and genetic background (Figure 4 and Figure 5). Male and female 50% cSOD activity flies lived longer than the cSOD-nulls (Figure 2). Furthermore, 50% cSOD activity females lived longer than males, although the magnitude of the sexual dimorphism varied across genetic backgrounds (Figure 4). Notably, while a comparable trend of longer-lived females was also seen in the 0% cSOD activity flies, *post hoc* analyses indicated that there was no significant difference in the longevity of the cSOD-nulls across sex or genetic background (Figure S4 in File S1), possibly reflecting the severity of the cSOD knockout condition. However, the lack of modifying effects on the cSOD-nulls is phenotype-specific as there were significant sex and genetic background effects in other phenotypes (below).

**Figure 6:**
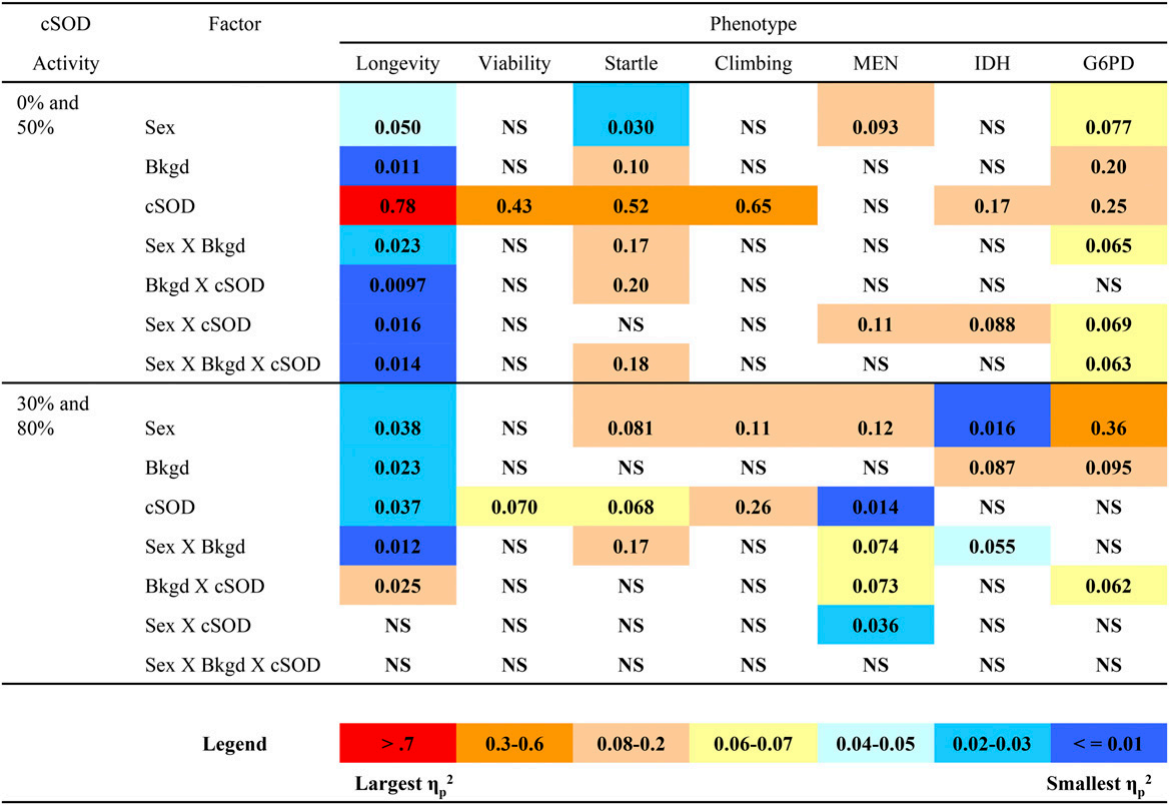
Partial eta squared (η*_p_*^2^) values for longevity, viability, negative geotaxis, countercurrent (climbing), malic enzyme (MEN), isocitrate dehydrogenase (IDH), and glucose-6-phosphate dehydrogenase (G6PD), calculated for each factor across cSOD (cytosolic copper/zinc superoxide dismutase) activity conditions. Larger values represent larger effects (oranges), and smaller values represent smaller effects (blues). NS, nonsignificant factors or interactions.

In the 30–80% comparison, cSOD activity still had a large effect on viability and longevity, with scores for both phenotypes lower at 30% cSOD than at 80% cSOD (Figure 2 and Figure 3). Consistent with the low cSOD comparison (above), viability was only affected by cSOD activity in the moderate cSOD comparison, while longevity was also sensitive to sex and genetic background (Figure S4 in File S1). Male and female 80% cSOD activity flies lived longer than 30% cSOD activity flies. As in the low-cSOD comparison, females lived longer than males across cSOD activities, with the magnitude of sexual dimorphism in longevity varying across genetic backgrounds (Figure 4). The effect of genetic background on longevity also varied across cSOD activities. Interestingly, while cSOD activity had a significant effect on life history phenotypes across comparisons, the effect size of cSOD on phenotypes was smaller when more cSOD activity was present (Figure 6).

### Locomotor phenotypes are most affected by cSOD activity and sex, but vary in sensitivity to genetic background

**Figure 7:**
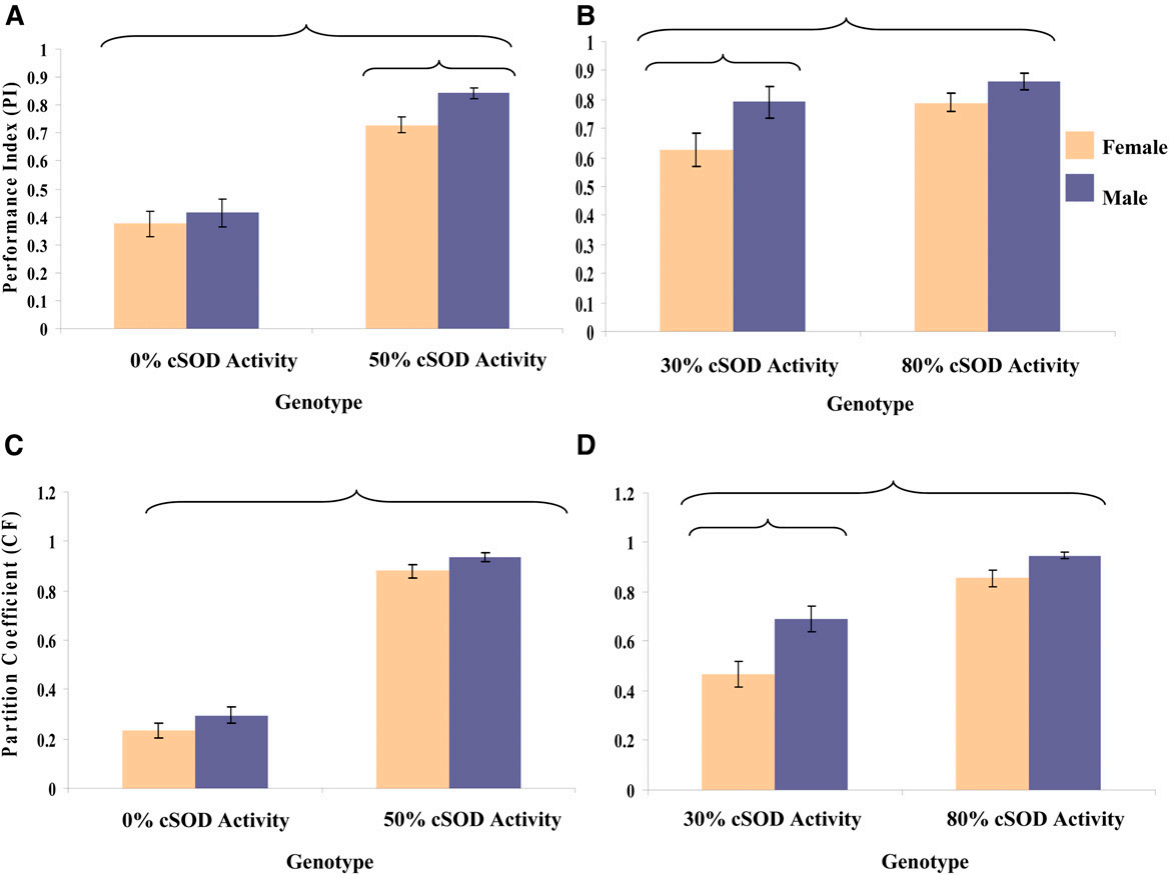
(A and B) Mean ± SEM of the negative geotaxis performance index (PI), and (C and D) mean ± SEM of the climbing partition coefficient (CF), for adult male and female flies pooled across the eight *DGRPi* genetic backgrounds within each genotype. ANOVA was used to test the factor effects with an α of 0.05. Partial eta squared (η*_p_*^2^) was calculated to quantify the effect of each factor. (A) cSOD Activity, *F*_1,156_ = 169.6, *P* < 0.0001, η*_p_*^2^ = 0.52; sex, *F*_1,156_ = 4.83, *P* = 0.0294, η*_p_*^2^ = 0.030. (B) cSOD Activity, *F*_1,100_ = 7.33, *P* = 0.008, η*_p_*^2^ = 0.0683; sex - *F*_1,100_ = 8.80, *P* = 0.0038, η*_p_*^2^ = 0.0809. (C) cSOD activity, *F*_1,269_ = 501.0, *P* < 0.0001, η*_p_*^2^ = 0.651. (D) cSOD activity, *F*_1,175_ = 61.6, *P* < 0.0001, η*_p_*^2^ = 0.260; sex - *F*_1,175_ = 20.8, *P* < 0.0001, η*_p_*^2^ = 0.106. Brackets enclose significantly different groups. cSOD, cytosolic copper/zinc superoxide dismutase.

As with the life history phenotypes, performance in both locomotor assays was lowest at 0% cSOD activity and higher at moderate cSOD activity (Figure 7). Locomotor ability was similar between the 50 and 80% cSOD activity groups, again indicating that only 50% cSOD activity was required to recover phenotypes to levels indistinguishable from WT. Also consistent with the life history phenotypes, cSOD activity had a larger effect than sex or genetic background on both geotaxis and countercurrent locomotion in the 0–50% comparison (Figure S5 and Figure S6 in File S1). Male and female 50% cSOD activity flies had better locomotor ability than the cSOD-nulls, across both phenotypes (Figure S7 in File S1). Interestingly, males had better geotaxic ability than females at both cSOD activities, though the magnitude of the sexual dimorphism varied across genetic backgrounds (Figure S5 in File S1). While there was no significant effect of sex on countercurrent locomotion in the low cSOD comparison, we noted a similar trend to that observed in geotaxis; males had better climbing ability than females (Figure S6 in File S1).

In the 30–80% comparison, cSOD activity had a large effect on geotaxis and countercurrent locomotion, where overall locomotor ability in both phenotypes at 30% cSOD was lower than at 80% cSOD (Figure 7). However, sex also had a significant effect; males had better locomotor ability than females in both phenotypes, across cSOD activities (Figure S7 in File S1). This difference is consistent with earlier studies (Long and Rice 2007), and potentially reflects differences in life history strategy between sexes. In geotaxis, but not countercurrent, the magnitude of the sexual dimorphism in locomotor ability varied across genetic backgrounds (Figure S5 and Figure S6 in File S1). Interestingly, genetic background had a larger effect on geotaxis in the 0–50% comparison than in the 30–80% comparison (trend discussed below; Figure 6 and Figure S7 in File S1). In contrast, the opposite trend was observed in life history and countercurrent locomotion (even if the effect was not significant).

In calculating the CF for countercurrent locomotion we binned flies into three classes: poor, moderate, and good climbers, essentially grouping flies with “similar” performance. To determine whether this binning was masking sex or genetic background effects, we recalculated the CF values using the vials as single classes (eight potential classes instead of three). This unbinned analysis also indicated no significant genetic background effects. The unbinned analysis indicated a significant effect of sex in the 0–50% comparison, and the trend of males having better climbing ability than females was observed in both analyses. Overall, binning seemed to have a limited effect on the analyses.

### NADP(H) enzymes have variable responses to cSOD activity, sex, and genetic background

**Figure 8:**
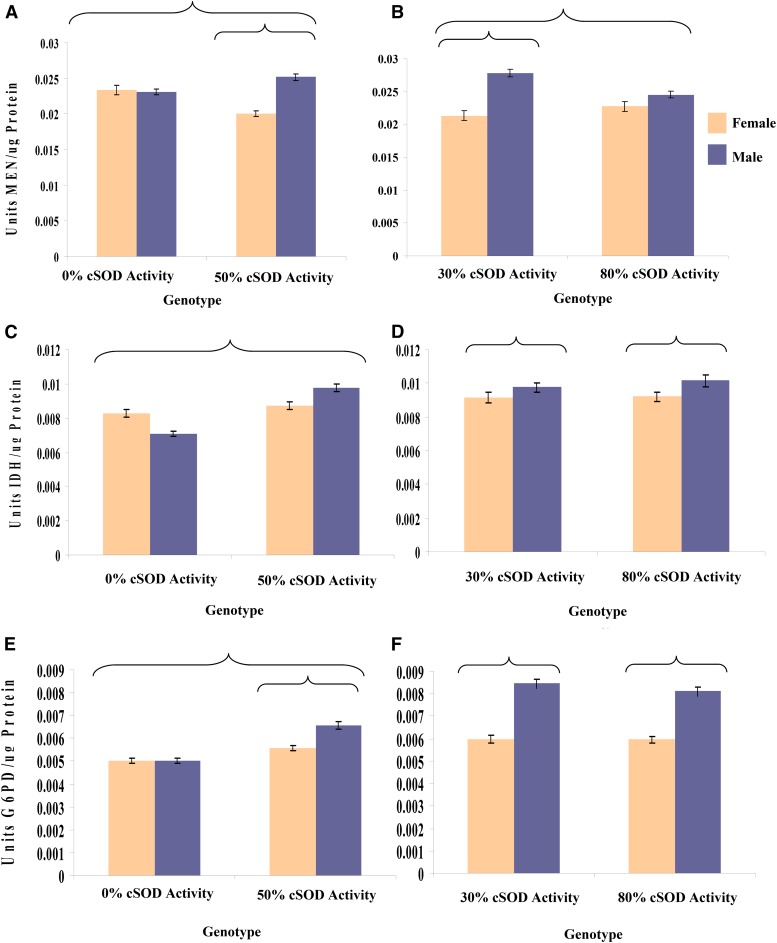
(A and B) Mean ± SEM of MEN activity; (C and D) mean ± SEM of the IDH activity; and (E and F) mean ± SEM of G6PD activity, standardized by protein concentration (micrograms per milliliter) for adult male and female flies pooled across the eight *DGRPi* genetic backgrounds within each 3rd chromosome genotype. ANOVA was used to test the factor effects with an α of 0.05. Partial eta squared (η*_p_*^2^) was calculated to quantify the effect of each factor. (A) Sex-by-cSOD activity, *F*_1,265_ = 31.8, *P* < 0.0001, η*_p_*^2^ = 0.110. (B) Sex-by-cSOD activity, *F*_1,280_ = 10.5, *P* = 0.0013, η*_p_*^2^ = 0.0363. (C) Sex-by-cSOD activity, *F*_1,265_ = 25.5, *P* < 0.0001, η*_p_*^2^ = 0.0879. (D) Sex, *F*_1,280_ = 4.58, *P* = 0.0333, η*_p_*^2^ = 0.0161. (E) Sex-by-cSOD activity, *F*_1,265_ = 19.6, *P* < 0.0001, η*_p_*^2^ = 0.0690. (F) Sex, *F*_1,280_ = 155.2, *P* < 0.0001, η*_p_*^2^ = 0.357. Brackets enclose significantly different groups. cSOD, cytosolic copper/zinc superoxide dismutase; *DGRPi*, ; G6PD, glucose-6-phosphate dehydrogenase; IDH, isocitrate dehydrogenase; MEN, malic enzyme.

**Figure 9:**
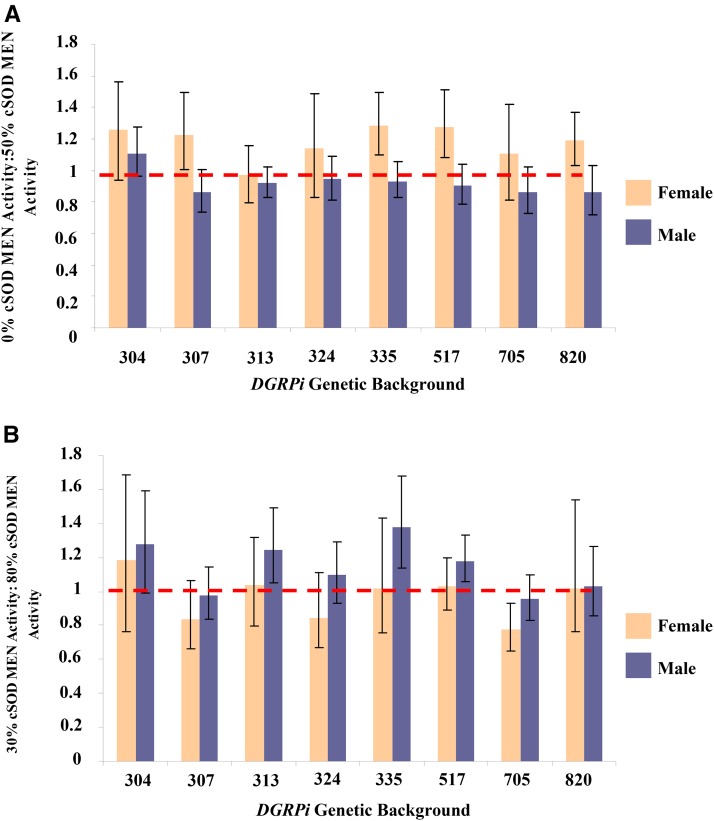
Mean ± SEM of the ratio of low cSOD: high cSOD activity MEN activity standardized by protein concentration (micrograms per milliliter) for adult male and female flies across the eight *DGRPi* genetic backgrounds. Values closer to 1 indicate more similar enzyme activities at the levels of cSOD activity being compared, and C.I.s are at 95% and represent the ratio of the low cSOD activity and high cSOD activity SEMs. (A) 0% cSOD activity: 50% cSOD activity: Female: lowest ratio 313, 0.796 < 0.970 < 1.16; highest ratio 335, 1.09 < 1.28 < 1.50; Male: lowest ratio 705, 0.730 < 0.863 < 1.02; highest ratio 304 - 0.963 < 1.11 < 1.28. (B) 30% cSOD activity: 80% cSOD activity: Female: lowest ratio 705, 0.646 < 0.777 < 0.929, highest ratio 304, 0.762 < 1.18 < 1.68; Male: lowest ratio 705, 0.827 < 0.956 < 1.10; highest ratio 335, 1.14 < 1.38 < 1.68. ANOVA was used to test the factor effects with an α of 0.05. Partial eta squared (η*_p_*^2^) was calculated to quantify the effect of each factor. (A) Sex-by-cSOD activity, *F*_1,265_ = 32.8, *P* < 0.0001, η*_p_*^2^ = 0.110. (B) Sex-by-genetic background, *F*_7,280_ = 3.18, *P* = 0.003, η*_p_*^2^ = 0.0736; Genetic background-by-cSOD activity, *F*_7,280_ = 3.16, *P* = 0.0031, η*_p_*^2^ = 0.0733; sex-by-cSOD activity, *F*_1,280_ = 10.5, *P* = 0.0013, η*_p_*^2^ = 0.0364. cSOD, cytosolic copper/zinc superoxide dismutase; *DGRPi*, ; MEN, malic enzyme.

The NADP(H) enzymes also responded to cSOD activity levels, but in contrast to the life history and locomotor phenotypes, which require 50% cSOD activity to recover WT phenotypes, 30% cSOD activity was sufficient to restore the biochemical phenotypes to WT activities (Figure 8). Consistent with the life history and locomotor phenotypes, the magnitude of sexual dimorphism in NADP(H) enzyme activities varied at 0 and 50% cSOD activities. However, in contrast to the previous phenotypes, the pattern of change differed between the sexes (Figure 6). In males, NADP(H) enzyme activities were all lower at 0% cSOD activity than at 50% cSOD activity, while in females the pattern varied across NADP(H) enzymes (Figure 9; Figure S8 and Figure S9 in File S1). Males tended to have higher NADP(H) enzyme activity than females, as long as some cSOD activity was present (Figure S10 in File S1). Strikingly, only G6PD activity was modified by genetic background in the 0–50% comparison, with the effect of genetic background varying between males and females across cSOD activities (Figure S9 in File S1).

In addition, and again comparable to life history and locomotor phenotypes, the effect of cSOD activity on the biochemical phenotypes tended to be smaller in the 30–80% comparison than the 0–50% comparison (Figure 6). Consistent with the 0–50% comparison (above), males tended to have higher activity than females as long as there was some cSOD activity (Figure 8). This consistent pattern suggests sex-specific sensitivity to variation in NADP(H) enzyme genotype in which males are more sensitive to differences in NADP(H) enzyme genotypes than females (Merritt *et al.* 2009). Interestingly, examination of gene expression data for cSOD, MEN, IDH, and G6PD, using the modMine function of ModENCODE, showed no indication of sex-specific expression at the whole organism level (Contrino *et al.*, 2012) in the lines available through FlyBase (data not shown). Overall, the biochemical phenotypes varied in their response to cSOD activity and genetic background in the 30–80% comparison (Figure 9; Figure S8 and Figure S9 in File S1). To determine if the biochemical results were simply a function of the covariate used (protein concentration), we repeated the biochemical analyses using fly mass and protein concentration as covariates and found essentially identical results to those using protein concentration alone (data not shown).

## Discussion

### Magnitudes of phenotypic responses vary across cSOD activities, sexes, and genetic backgrounds

Differences in cSOD activity consistently resulted in substantial changes in life history, locomotor, and biochemical phenotypes, and these large-scale differences were significantly modified by genetic background and sex. Previous research in *D. melanogaster* has shown that complete loss of cSOD activity modifies a broad suite of phenotypes, but that as little as 50% of normal cSOD activity is generally sufficient to fully restore WT phenotypes (Phillips *et al.* 1989; Parkes *et al.* 1998b; Sun and Tower 1999; Pasyukova *et al.* 2000; Woodruff *et al.* 2004; Bernard *et al.* 2011; Jones and Grotewiel 2011; Knee *et al.* 2013). However, these studies have predominantly used single, isogenic backgrounds, even though complex phenotypes are generally influenced by multiple genes (Laurie-Ahlberg *et al.* 1982; Leips and Mackay 2000; Spencer *et al.* 2003; Jordan *et al.* 2007; Rzezniczak and Merritt 2012). Sex and genetic background both significantly modify the cSOD effects that we demonstrate, but interestingly these modifications are relatively small compared to the effect of cSOD. Across the eight backgrounds we analyzed, no one background eliminated, or even strongly ameliorated or exaggerated, any of the cSOD phenotypes.

We expected life history and locomotor phenotypes to be a function of a larger number of genetic and environmental interactions than the biochemical phenotypes; therefore, we expected to see greater sensitivity in life history and locomotor phenotypes to changes in cSOD activity, sex, and genetic background. Instead, all of the phenotypes showed similar sensitivity to all three factors. However, life history and locomotor phenotypes were more sensitive to smaller differences in cSOD activity than biochemical phenotypes, possibly supporting our expectation that these phenotypes are more complex than the biochemical activities. Interestingly, a lower threshold of cSOD activity was required to recover the biochemical phenotypes to WT, although it is unclear if this difference reflects a fundamental difference between phenotype classes.

### The absence of cSOD activity has the largest effect on all phenotypes examined

We also expected that cSOD activity would be the most important factor controlling phenotypes at low cSOD activity, reflecting the oxidative stress and broad-reaching metabolic changes caused by the loss-of-function of a key antioxidant defense enzyme. Consistent with our expectation, phenotypes were most altered in cSOD-nulls, and loss of cSOD activity had a larger effect than sex or any of the backgrounds. Consistent with earlier studies (*e.g.*, Seto *et al.* 1990) we found that phenotypes were recovered to approximately WT levels by 50% cSOD activity, supporting the idea that this amount of cSOD activity is generally sufficient to overcome paraquat toxicity. In addition, while the magnitude of the phenotypic responses differed, more variation was attributable to cSOD activity in the 0–50% comparison than in the 30–80% comparison.

Life history and locomotor phenotypes were significantly compromised in flies with no cSOD activity, with phenotypic differences reflecting the amount of cSOD activity (Figure S4 in File S1), consistent with other studies (Parkes *et al.* 1998b; Sun and Tower 1999; Jones and Grotewiel 2011). Interestingly, the NADP(H) enzymes each responded differently to changes in cSOD activity. These unique responses are in contrast to our earlier study (Bernard *et al.* 2011), in which we found similar reductions in the enzyme activities, possibly reflecting the different backgrounds used in these two studies.

The sensitivity of the biochemical phenotypes to changes in cSOD activity, sex, and genetic background, suggests that these “simple” phenotypes are more complicated than we expected or at least that their connection to cSOD is. Originally, we classified the biochemical phenotypes as “proximal” (phenotypes close to the mutation), and the life history and locomotor phenotypes as “distal” (phenotypes far from the mutation). As distal phenotypes are further downstream of the mutation, we expected them to be a function of a larger number of genetic and environmental interactions than the proximal phenotypes. However, interactions between MEN, IDH, and G6PD (Merritt *et al.* 2009; Rzezniczak and Merritt 2012), and between these NADP(H) enzymes and cSOD [*e.g.*, in Bernard *et al.* (2011) and results from this study], indicate that ties between cSOD and the NADP(H) enzymes are complex and influenced by multiple genes. Clearly, these biochemical phenotypes are not as proximal to cSOD as we envisioned and the simple “proximal” and “distal” classifications originally assigned do not accurately reflect the nature of the phenotypes measured. However, the fact that the biochemical phenotypes are recovered to WT by a lower threshold of cSOD than the life history and locomotor phenotypes likely reflects differences in the relative influence of cSOD on these types of phenotypes. Whether the paths of interaction connecting cSOD, modifying loci, and the biochemical phenotypes are more or less complex than those involved in modifying life history and locomotor traits remains unclear, and further research including enzymes more directly connected with cSOD, such as catalase (Michiels *et al.* 1994), is needed.

The strength of interaction between cSOD and each phenotype likely reflects the degree of connectedness between cSOD and the molecular mechanisms of each phenotype, presumably indirectly through the role of cSOD and NADP(H) in the antioxidant defense network (Phillips *et al.* 1989; Bernard *et al.* 2011). The more closely phenotypes are linked to ROS metabolism, the greater the effects of differences in cSOD activity on the phenotypes. Further, different degrees of connectedness should result in, or be a component of, different cSOD activity thresholds required to recover WT phenotypes. These interactions could be through NADP(H) consumption, or the accumulation of superoxide-inducing oxidative stress. In either scenario, complete loss of cSOD activity should have the greatest effect, with this extreme condition limiting the modifying effects of sex or genetic background, consistent with our results.

### Sex has a larger effect on phenotypes at higher cSOD activity

cSOD and associated phenotypes have primarily been studied in males (Parkes *et al.* 1998a; Bernard *et al.* 2011; Knee *et al.* 2013), with the exception of some sex-specific phenotypes (*e.g.*, male and female fertility; Parkes *et al.* 1998b). This exclusion reflects a larger bias against inclusion of female subjects in biological studies (Miller 2012; Klein *et al.* 2015; Parikh 2015). The predominant use of male subjects reflects the perception that females are more variable, and therefore poorer research subjects, than males. Our results do not support this perception. It is possible that the increased metabolic burden imposed by reproductive effort in females, relative to males, could increase variability in life history phenotypes (*e.g.*, viability) among females, potentially confounding analysis of such phenotypes. However, Figure 2 illustrates the relative variability in longevity between males and females observed in this study and clearly shows that females are not substantially more variable than males. By expressing the SEMs for longevity in both sexes as a percentage of the means themselves, the relative longevity data reveal that variability is very similar between the sexes: 1.2–2.4% of the mean in females, and 0.9–1.9% in males. The other six phenotypes showed a similar pattern; females were not substantially more variable than males. It is important to note that assays in this study were performed on mated flies, so any increased variability in females resulting from variability in reproductive effort should be apparent, yet there is no evidence in our data to support the notion that females are more phenotypically variable than are males. Given the sex-specific differences that we documented, and the importance of understanding full phenotypic effects, not simply male-specific effects, it is crucial that traits be studied in both males and females.

Sex-specific phenotypic differences have been documented in *D. melanogaster* and many other organisms, including differences in some of the phenotypes we quantified (*e.g.*, Pasyukova *et al.* 2000; Spencer *et al.* 2003; Jordan *et al.* 2007; Merritt *et al.* 2009). We observed significant sexual dimorphism in longevity, geotaxis, countercurrent locomotion, and NADP(H) enzyme activities, indicating that such differences are widespread. One qualifying note: male and female flies in this experiment differed in genotype at the X chromosome; males have only the *w^+^* X chromosome, females have the *w^+^* X and the *w^1118^* X. It is possible, therefore, that some dominant effects from the *w^1118^* chromosome could influence our results. However, the fact that the sexual dimorphism we observed is consistent with that documented in the literature (Spencer *et al.* 2003; Jordan *et al.* 2007; Merritt *et al.* 2009) suggests that the differences observed are not only a function of differences in the X chromosome genotype.

We did find that cSOD activity itself was sexually dimorphic, albeit only in the 30–80% comparison. Sexual dimorphism in longevity has been reported in flies overexpressing cSOD, possibly reflecting genetic background or sex-specific activity of the Gal4 activator and/or the cSOD transgene employed in those studies (Spencer *et al.* 2003). The sex-specific disparity in assayed cSOD activity we observe may similarly reflect sexual dimorphism in expression from the *T*5 cSOD transgene. As no sexual dimorphism in cSOD activity or in gene expression exists at the native *D. melanogaster cSod* locus (Kopp *et al.* 2003; Gnad and Parsch 2006), it seems likely that the sexual dimorphism in cSOD activity in the 30–80% comparison reflects some feature of the transgene, possibly reflecting local genomic environment, and is not a general feature of cSOD.

### There were substantial differences in sensitivity to genetic background across the phenotypes examined

Differences in phenotypes across genetic backgrounds, “line effects,” are common, driven by variation at modifying loci across the genome. Previous research has shown that some life history traits differ across genetic backgrounds (*e.g.*, Fry *et al.* 1998; Spencer *et al.* 2003), while others do not (de Visser *et al.* 2003; Fry 2008). In our study, longevity and viability differed in sensitivity to genetic background, with only longevity being significantly affected by genetic background perturbation (Figure S4 in File S1). Fitness and longevity vary across the parent DGRP lines (Mackay *et al.* 2012) and we expected variation in the lines we constructed, but likely somewhat lesser amounts reflecting the inclusion of only variation at the 2nd chromosome in our study. The fact that we found longevity, but not viability, to be sensitive to genetic background, suggests that not only are the genetic networks for viability and longevity independent, but that the network for viability is more robust than longevity.

Two complementary locomotor assays were performed to detect small differences in the effect of genetic background on locomotion: negative geotaxis, and countercurrent locomotion. While genetic background effects were detected in geotaxis across cSOD comparisons, the effects were smaller in the 30–80% comparison than in the 0–50% comparison (Figure 6), consistent with the reduction in resolving power we hypothesized could result from modifying the climbing height in the geotaxis assay (see *Materials and Methods*). Interestingly, countercurrent locomotion was less sensitive to genetic background or sex than was geotaxis (Figure S7 in File S1). The countercurrent assay is more of an endurance, rather than reactive, assay than the geotaxis assay (Jones and Grotewiel 2011), and the smaller effects of background may reflect a particular deficit in locomotor stamina in cSOD-null flies. In general, the two locomotor assays responded differently to variation in genetic background, suggesting that these locomotor phenotypes have different genetic mechanisms, consistent with previous research (Jordan *et al.* 2007, 2012).

MEN, IDH, and G6PD are part of an interconnected NADP(H) network, in which changes in activity of one enzyme cause changes in activities of the other enzymes, likely through pools of the shared NADP(H) cofactor (Merritt *et al.* 2009; Bernard *et al.* 2011; Rzezniczak and Merritt 2012; Rzezniczak *et al.* 2012). Previously, similar responses in MEN, IDH, and G6PD to differences in cSOD activity were observed in a single genetic background (Bernard *et al.* 2011). We demonstrate here that these interactions are, in fact, sensitive to genetic background, and that each enzyme differs in this sensitivity (Figure S10 in File S1). While all these enzymes share the NADP(H) cofactor, each component of this network responds differently to genetic, metabolic, and environmental changes, and interactions across the network are complex and not simply compensatory (Rzezniczak and Merritt 2012; Merritt *et al.* 2009).

### None of the genetic backgrounds demonstrated consistent modifications of the cSOD phenotypes

We selected DGRP lines for second chromosome extractions specifically to maximize differences in our phenotypes, selecting high and low lines for each phenotype, but observed only relatively small modifications of the cSOD effects by background. However, complex phenotypes are controlled by multiple genes with phenotypic differences likely driven by variation across the genome (none of the phenotypes have been fully mapped), and the lines we created will only reveal dominant modifiers at 2nd chromosome loci. Extracting “high” and “low” line chromosomes from the parent lines altered their genetic context that may have modified the interactions that led to their high or low line classification. Further, we were constrained to only assaying 2nd chromosome heterozygotes, preventing us from observing any recessive interactions. One general conclusion across all the phenotypes is that genetic background effects were smallest in the cSOD-nulls, suggesting that this extreme condition limits these effects, but others [reviewed in Chari and Dworkin (2013)] have demonstrated background effects across all types of mutations. This contrast suggests that the presence and magnitude of background effects is both gene- and phenotype-specific.

Even with the caveat of the restriction to dominant modifiers, we were surprised that no background substantially suppressed or enhanced the cSOD-null phenotypes. Genetic variation at the 2nd chromosome background modified, but did not extensively change, the phenotypic variation that we observe. The fact that the parent DGRP lines show substantial variation for each phenotype indicates that the genetic variation present is capable of modifying the phenotypes. Thus, the lack of large-scale variation in our derived lines likely reflects a complex genomic architecture in background effects, suggesting that these effects are themselves complex and involve dominant and recessive interactions between loci across the genome.

### Conclusions

The cSOD-null syndrome is driven by the absence of cSOD activity, but is sensitive to, and modified by, sex and genetic background. While smaller in magnitude than the cSOD effects, background and sex did significantly affect phenotypes, including notable sex-specific effects, the magnitude of genetic background effects vary strikingly with the level of cSOD activity and phenotype. Consistent with this phenotype-specific sensitivity, the threshold amount of cSOD activity required to recover a phenotype to WT also differs, with life history and locomotor phenotypes having a higher threshold cSOD activity than the biochemical phenotypes. Surprisingly, no genetic backgrounds were found that resulted in large-scale enhancement or suppression of cSOD-dependent phenotypes. Finally, our results indicate that male and female flies substantially differ in their phenotypic responses to some factors, reinforcing the point that both male and female subjects, of any species, need to be assayed to understand biochemical and physiological processes.

## Supplementary Material

Supplemental material is available online at www.g3journal.org/lookup/suppl/doi:10.1534/g3.117.043836/-/DC1.
