# Supplementary material for: Sex and Genetic Background Influence Superoxide Dismutase (cSOD)-Related Phenotypic Variation in Drosophila melanogaster
Source: G3 (Bethesda). 2017 Jun 17;7(8):2651–64. doi: 10.1534/g3.117.043836 (PMC5555470; doi:10.1534/g3.117.043836)
Supplement: Supplementary file 1 [file 2651File001.doc]

# Supplemental Figures


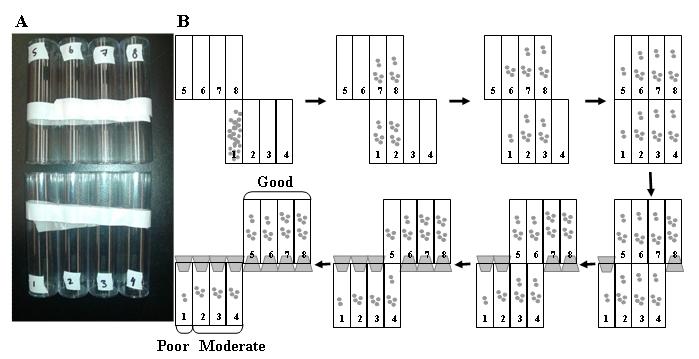


**Figure S1 Coutercurrent Assay Apparatus:** Photo A) and schematic diagram B) showing the countercurrent apparatus as modified by Peterson *et al*. (2013). Flies enter the apparatus at vial 1, in the bottom vial set, as indicated by the grey circles, and vial 8, in the top vial set, is inverted over vial 1 (Peterson *et al*. 2013). The vials are tapped down and the flies are given one minute to climb (Peterson *et al*. 2013). Following one minute the top set of vials was shifted over, and the flies in the top vials were tapped down, and allowed to climb for one minute (Peterson *et al*. 2013). This procedure was repeated seven times, and vials that were not opposed by other vials were plugged to prevent flies from escaping (Peterson *et al*. 2013). Fly climbing ability was designated poor, moderate, or good dependent on the vial number the flies ended up in, as marked in the figure (Peterson *et al*. 2013).


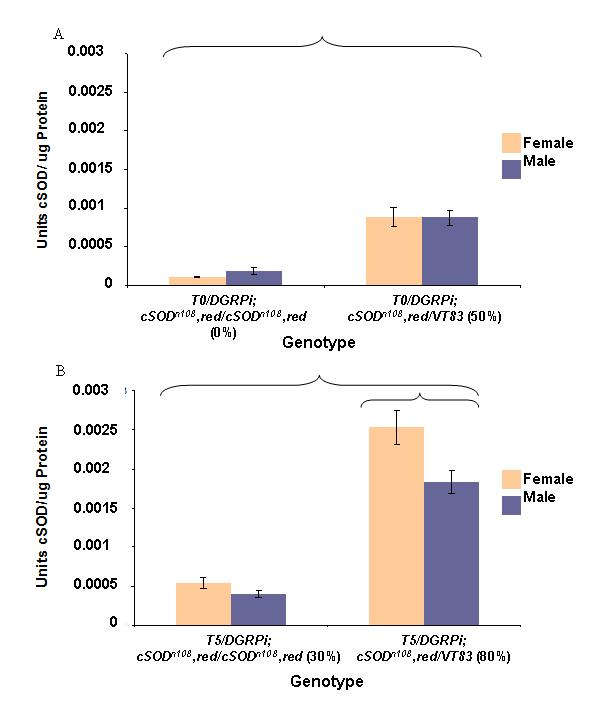


**Figure S2 T0 and T5 cSOD Activity Backgrounds Pooled:** Mean ± SEM of the measured cSOD activities for adult male and adult female flies pooled across the eight *DGRPi* genetic backgrounds within each 3^rd^ chromosome genotype. A) 0% cSOD activity: Female - 0.000106 ± 0.000017 U/μg, Male - 0.000192 ± 0.000048 U/μg ; 50% cSOD activity: Female - 0.000883 ± 0.00012 U/μg, Male - 0.000875 ± 0.00010 U/μg B) 30% cSOD activity: Female - 0.000546 ± 0.000069 U/μg, Male - 0.000408 ± 0.000045 U/μg; 80% cSOD Activity: Female - 0.00253 ± 0.00021 U/μg, Male - 0.00184 ± 0.00015 U/μg. Analysis of variance was used to test the factor effects with an alpha of 0.05. Partial eta squared (η_p_^2^) was calculated to quantify the effect of each factor. A) cSOD Activity - F_1,127_ = 84.1, p < 0.0001, η_p_^2^ = 0.398 B) cSOD Activity - F_1,124_ = 157.6, p < 0.0001, η_p_^2^ = 0.560, Sex - F_1,124_ = 9.65, p = 0.0023, η_p_^2^ = 0.0722. Brackets enclose significantly different groups.


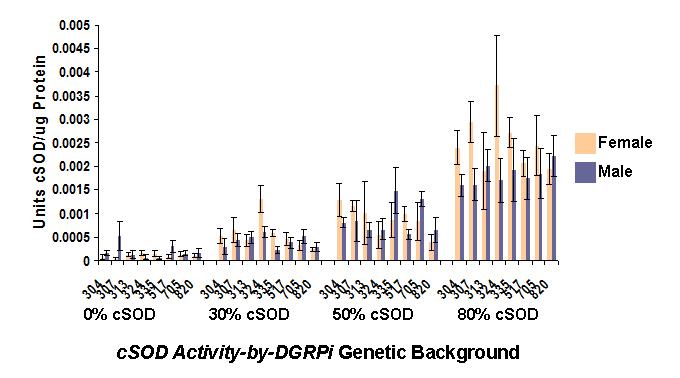


**Figure S3 T0 and T5 cSOD Activity Background Trends:** Summary of the mean ± SEM background responses for cSOD activity showing the trend of cSOD activity response to changes in genotype, with cSOD activity measured as U/mL standardized by protein concentration (ug/mL). 0% cSOD activity - Female: Low - (307) 0.0000310 ± 0.000021, High - (324) 0.000156 ± 0.000055; Male: Low - (335) 0.0000520 ± 0.000036, High - (313) 0.000133 ± 0.000094. 30% cSOD activity - Female: Low - (820) 0.000238 ± 0.000057, High - (324) 0.00130 ± 0.00028; Male: Low - (335) 0.000226 ± 0.000084, High - (324) 0.000607 ± 0.00012. 50% cSOD activity - Female: Low - (820) 0.000390 ± 0.00017, High - (304) 0.00129 ± 0.00035; Male: Low - (517) 0.000560 ± 0.00011, High - (335) 0.00149 ± 0.00050. 80% cSOD activity - Female: Low - (313) 0.00191 ± 0.00081, High - (324) 0.00371 ± 0.0011; Male: Low - (304) 0.00160 ± 0.00024, High - (820) 0.00222 ± 0.00045. Analysis of variance was used to test the factor effects with an alpha of 0.05. Partial eta squared (η_p_^2^) was calculated to quantify the effect of each factor. 0% and 50% cSOD Activity: cSOD Activity - F_1,127_ = 84.1, p < 0.0001, η_p_^2^ = 0.398. 30% and 80% cSOD Activity: cSOD Activity - F_1,124_ = 157.6, p < 0.0001, η_p_^2^ = 0.560; Sex - F_1,124_ = 9.65, p = 0.0023, η_p_^2^ = 0.0722. Brackets enclose significantly different groups.


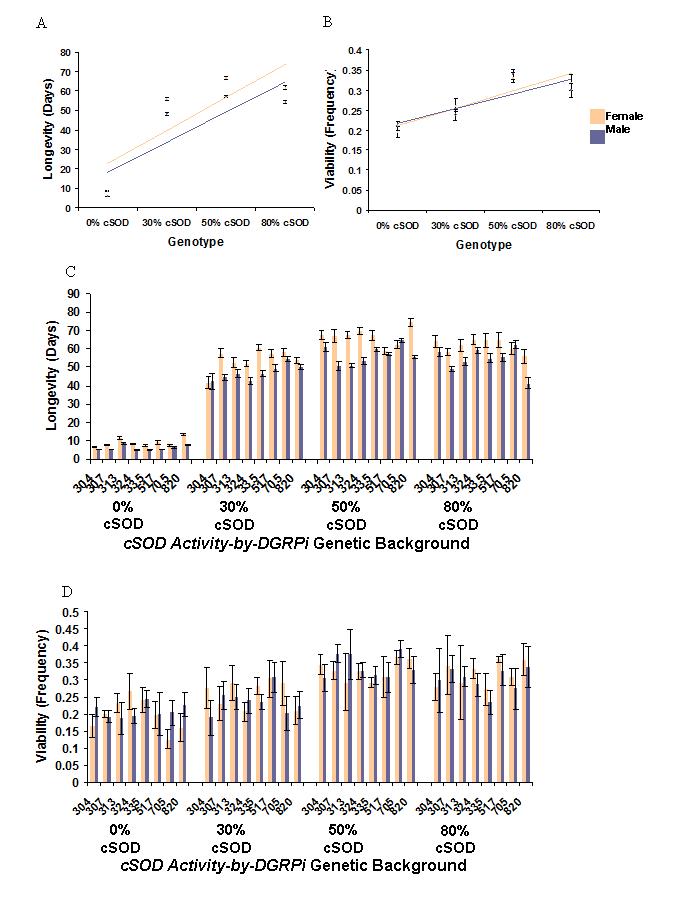


**Figure S4 T0 and T5 Summary of Life History Trends:** Analysis of variance was used to test the factor effects with an alpha of 0.05. Partial eta squared (η_p_^2^) was calculated to quantify the effect of each factor. Summary of the mean ± SEM responses pooled across backgrounds for longevity A) and viability B) across the four levels of cSOD activity surveyed showing the trend of phenotypic response to changes in cSOD activity. Summary of the mean ± SEM background responses for longevity C) and viability D) across the four levels of cSOD activity surveyed showing the trend of phenotypic response to changes in cSOD activity.


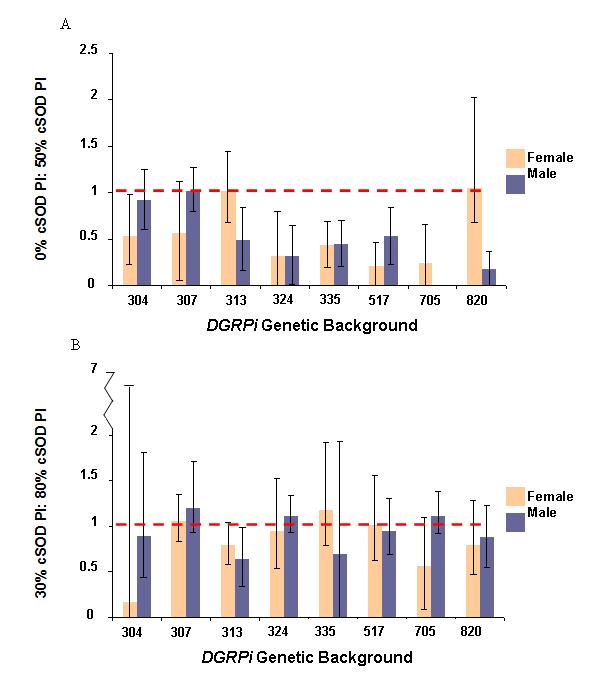


**Figure S5 T0 and T5 Negative Geotaxis Background Ratios:** Mean ± SEM of the ratio of low cSOD: high cSOD activity negative geotaxis performance index (PI) values for adult male and female flies across the eight *DGRPi* genetic backgrounds. Values closer to 1 indicate more similar PI values at the levels of cSOD activity being compared, and confidence intervals (CI) are at 95% and represent the ratio of the low cSOD activity and high cSOD activity SEMs. A) 0% cSOD activity: 50% cSOD activity: Female: Lowest Ratio 517 - 0 < 0.207 < 0.461, Highest Ratio 820 - 0.676 < 1.05 < 2.02; Male: Lowest Ratio 705 - 0, Highest Ratio 307 - 0.766 < 1.01 < 1.26 B) 30% cSOD activity: 80% cSOD activity: Female: Lowest Ratio 304 - 0 < 0.167 < 6.91 (90% CI), Highest Ratio 335 - 0.790 < 1.17 < 1.92; Male: Lowest Ratio 313 - 0.345 < 0.637 < 0.983, Highest Ratio 307 - 0.928 < 1.20 < 1.80. Analysis of variance was used to test the factor effects with an alpha of 0.05. Partial eta squared (η_p_^2^) was calculated to quantify the effect of each factor. A) Sex-by-Genetic Background-by-cSOD Activity - F_1,156_ = 4.95, p < 0.0001, η_p_^2^ = 0.182 B) cSOD Activity - F_1,100_ = 7.33, p = 0.008, η_p_^2^ = 0.0683, Sex-by-Genetic Background - F_1,100_ = 3.02, p = 0.0064, η_p_^2^ = 0.174.


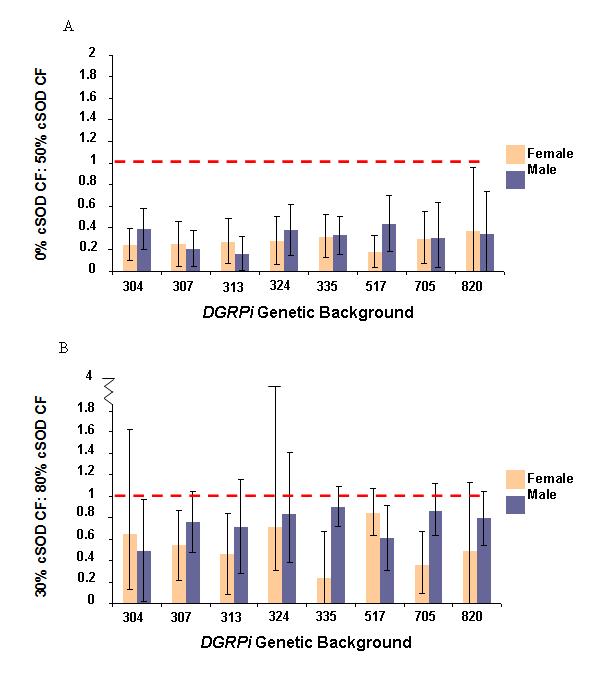


**Figure S6 T0 and T5 Countercurrent Background Ratios:** Mean ± SEM of the ratio of low cSOD: high cSOD activity climbing ability partition coefficient (CF) for adult male and female flies across the eight *DGRPi* genetic backgrounds. Values closer to 1 indicate more similar CF values at the levels of cSOD activity being compared, and confidence intervals (CI) are at 95% and represent the ratio of the low cSOD activity and high cSOD activity SEMs. A) 0% cSOD activity: 50% cSOD activity - Female: Lowest Ratio 517 - 0.0333 < 0.175 < 0.335, Highest Ratio 820 - 0 < 0.367 < 0.957; Male: Lowest Ratio 313 - 0.00850 < 0.164 < 0.321, Highest Ratio 517 - 0.186 < 0.437 < 0.706 B) 30% cSOD activity: 80% cSOD activity - Female: Lowest Ratio 335 - 0 < 0.235 < 0.673, Highest Ratio 517 - 0.632 < 0.839 < 1.07; Male: Lowest Ratio 304 - 0.0162 < 0.492 < 0.974, Highest Ratio 335 - 0.720 < 0.902 < 1.08. Analysis of variance was used to test the factor effects with an alpha of 0.05. Partial eta squared (η_p_^2^) was calculated to quantify the effect of each factor. A) cSOD Activity - F_1,269_ = 501.0, p < 0.0001, η_p_^2^ = 0.651 B) Sex - F_1,175_ = 20.8, p < 0.0001, η_p_^2^ = 0.106, cSOD Activity - F_1,175_ = 61.6, p < 0.0001, η_p_^2^ = 0.260.


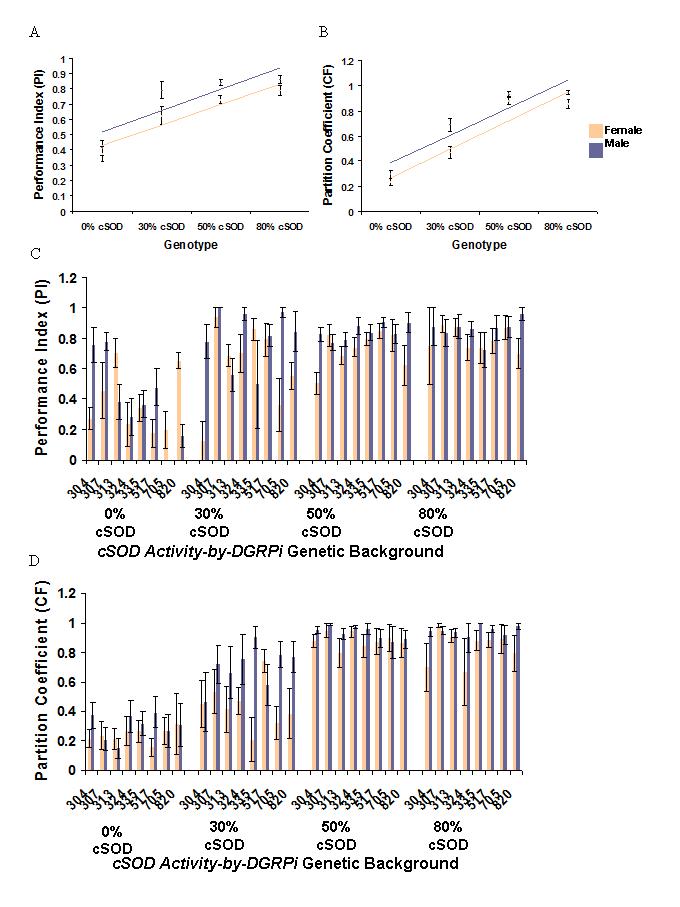


**Figure S7 T0 and T5 Summary of Locomotor Trends:** Analysis of variance was used to test the factor effects with an alpha of 0.05. Partial eta squared (η_p_^2^) was calculated to quantify the effect of each factor. Summary of the mean ± SEM responses pooled across backgrounds for negative geotaxis A) and climbing ability B) across the four levels of cSOD activity surveyed showing the trend of phenotypic response to changes in cSOD activity. Summary of the mean ± SEM background responses for negative geotaxis C) and climbing ability D) across the four levels of cSOD activity surveyed showing the trend of phenotypic response to changes in cSOD activity.


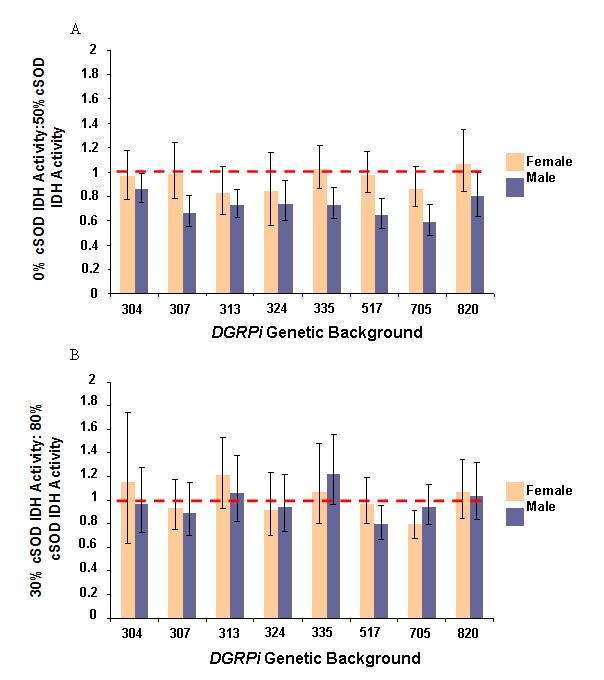


**Figure S8 T0 and T5 IDH Activity Background Ratios:** Mean ± SEM of the ratio of low cSOD: high cSOD activity IDH activity standardized by protein concentration (μg/mL) for adult male and female flies across the eight *DGRPi* genetic backgrounds. Values closer to 1 indicate more similar enzyme activties at the levels of cSOD activity being compared, and confidence intervals (CI) are at 95% and represent the ratio of the low cSOD activity and high cSOD activity SEMs. 0% cSOD activity: 50% cSOD activity: Female: Lowest Ratio 313 - 0.650 < 0.832 < 1.04, Highest Ratio 820 - 0.839 < 1.06 < 1.35; Male: Lowest Ratio 705 - 0.474 < 0.585 < 0.730, Highest Ratio 304 - 0.752 < 0.866 < 0.988. 30% cSOD activity: 80% cSOD activity: Female: Lowest Ratio 517 - 0.666 < 0.793 < 0.958, Highest Ratio 335 - 0.960 < 1.22 < 1.55; Male: Lowest Ratio 705 - 0.680 < 0.793 < 0.914, Highest Ratio 313 - 0.928 < 1.21 < 1.53. Analysis of variance was used to test the factor effects with an alpha of 0.05. Partial eta squared (η_p_^2^) was calculated to quantify the effect of each factor. A) Sex-by-cSOD - F_1,265_ = 25.5, p < 0.0001, η_p_^2^ = 0.0879 B) Sex-by-Genetic Background - F_7,280_ = 2.31, p = 0.0263, η_p_^2^ = 0.0547.


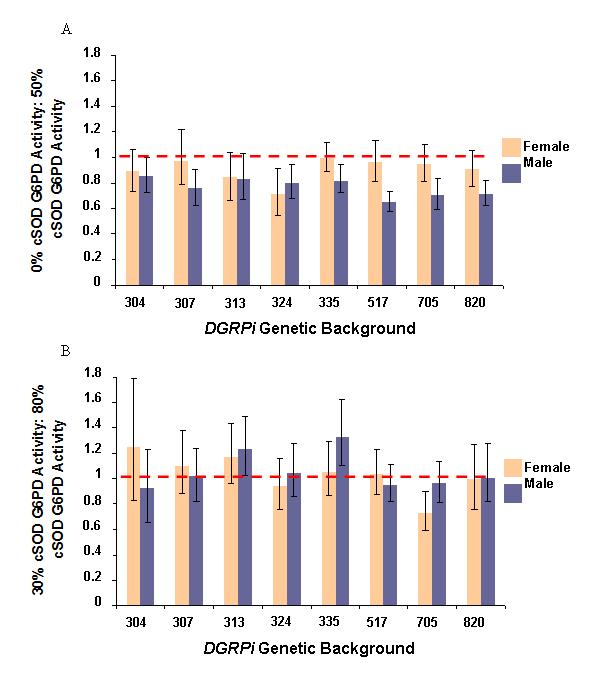


**Figure S9 T0 and T5 G6PS Activity Background Ratios:** Mean ± SEM of the ratio of low cSOD: high cSOD activity G6PD activity standardized by protein concentration (μg/mL) values for adult male and female flies across the eight *DGRPi* genetic backgrounds. Values closer to 1 indicate more similar enzyme activities at the levels of cSOD activity being compared, and confidence intervals (CI) are at 95% and represent the ratio of the low cSOD activity and high cSOD activity SEMs. A) 0% cSOD activity: 50% cSOD activity: Female: Lowest Ratio 324 - 0.542 < 0.715 < 0.912, Highest Ratio 335 - 0.893 < 0.999 < 1.12; Male: Lowest Ratio 517 - 0.575 < 0.649 < 0.738, Highest Ratio 304 - 0.721 < 0.849 < 1.00. B) 30% cSOD activity: 80% cSOD activity: Female: Lowest Ratio 705 - 0.586 < 0.722 < 0.896, Highest Ratio 304 - 0.825 < 1.24 < 1.80; Male: Lowest Ratio 304 - 0.655 < 0.924 < 1.23, Highest Ratio 335 - 1.10 < 1.33 < 1.62. Analysis of variance was used to test the factor effects with an alpha of 0.05. Partial eta squared (η_p_^2^) was calculated to quantify the effect of each factor. A) Sex-by-Genetic Background-by-cSOD Activity - F_7,265_ = 2.57, p = 0.0142, η_p_^2^ = 0.0635 B) Sex - F_1,280_ = 155.2, p < 0.0001, η_p_^2^ = 0.366, Genetic Background-by-cSOD Activity - F_7,280_ = 2.65, p = 0.0114, η_p_^2^ = 0.0621.


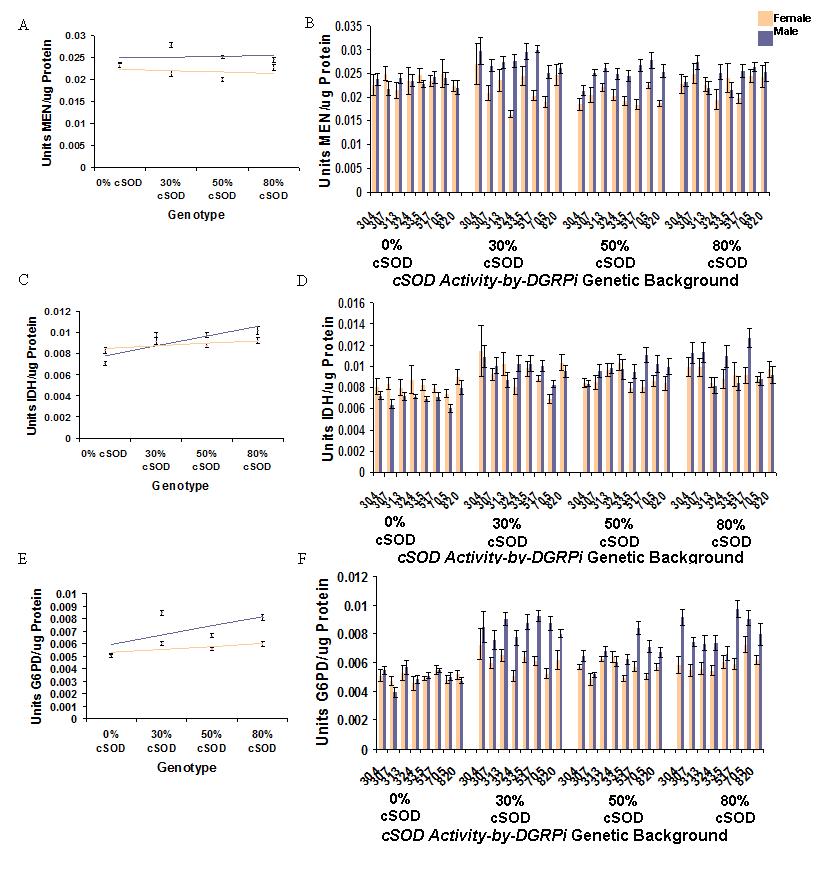


**Figure S10 T0 and T5 Summary of Biochemical Trends:** Analysis of variance was used to test the factor effects with an alpha of 0.05. Partial eta squared (η_p_^2^) was calculated to quantify the effect of each factor. Summary of the mean ± SEM responses pooled across backgrounds for MEN activity A) IDH activity C) and G6PD activity E) across the four levels of cSOD activity surveyed showing the trend of phenotypic response to changes in cSOD activity. Summary of the mean ± SEM background responses for MEN activity B) IDH activity D) and G6PD activity F) across the four levels of cSOD activity surveyed showing the trend of phenotypic response to changes in cSOD activity.

# Supplemental Tables

**Table S1: L**ongevity ANOVA

| Source | d.f. | MS | F | P | η_p_^2^ |
| --- | --- | --- | --- | --- | --- |
| T0 - 0% and 50% cSOD Activity Cross | | | | |  |
| Sex | 1 | 20926.2 | 116 | **<.0001** | 0.0497 |
| Background | 7 | 645.0571 | 3.59 | **0.0008** | 0.0111 |
| cSOD Activity | 1 | 1446024 | 8040 | **<.0001** | 0.783 |
| Sex X Background | 7 | 1317.286 | 7.33 | **<.0001** | 0.0225 |
| Background X cSOD Activity | 7 | 561.4857 | 3.12 | **0.0028** | 0.00972 |
| Sex X cSOD Activity | 1 | 6330 | 35.2 | **<.0001** | 0.0156 |
| Sex X Background X cSOD Activity | 7 | 788.1 | 4.38 | **<.0001** | 0.0136 |
| Error | 2227 | 179.8 |  |  |  |
|  |  |  |  |  |  |
| T5 - 30% and 80% cSOD Activity Cross | | | | |  |
| Sex | 1 | 20660.74 | 66.1 | **<.0001** | 0.0380 |
| Background | 7 | 1768.072 | 5.66 | **<.0001** | 0.0231 |
| cSOD Activity | 1 | 20267.86 | 64.9 | **<.0001** | 0.0373 |
| Sex X Background | 7 | 910.6223 | 2.91 | **0.0049** | 0.0120 |
| Background X cSOD Activity | 7 | 1884.536 | 6.03 | **<.0001** | 0.0246 |
| Sex X cSOD Activity | 1 | 23.43 | 0.0750 | 0.784 | 4.48E-05 |
| Sex X Background X cSOD Activity | 7 | 399.7026 | 1.28 | 0.257 | 0.00533 |
| Error | 1673 | 312.41 |  |  |  |

Raw longevity ANOVA outputs indicating significance of factors and their interactions, as well as the effect sizes of individual factors and interactions. Analysis of variance was used to test the factor effects with an alpha of 0.05. Effect sizes were calculated as partial eta squared (η_p_^2^). Bolded values denote significant factors in longevity determination.

Table S2: Viability ANOVA

| Source | d.f. | MS | F | P | η_p_^2^ |
| --- | --- | --- | --- | --- | --- |
| T0 - 0% and 50% cSOD Activity Cross | | | | |  |
| Sex | 1 | 0.00566827 | 0.856 | 0.356 | 0.00595 |
| Background | 7 | 0.001510739 | 0.228 | 0.978 | 0.0110 |
| cSOD Activity | 1 | 0.70608036 | 107 | **<.0001** | 0.427 |
| Sex X Background | 7 | 0.003158474 | 0.477 | 0.850 | 0.0228 |
| Background X cSOD Activity | 7 | 0.012290796 | 1.86 | 0.081 | 0.0833 |
| Sex X cSOD Activity | 1 | 0.00008999 | 0.0136 | 0.907 | 9.51E-05 |
| Sex X Background X cSOD Activity | 7 | 0.008188177 | 1.24 | 0.286 | 0.0571 |
| Error | 143 | 0.006619 |  |  |  |
|  |  |  |  |  |  |
| T5 - 30% and 80% cSOD Activity Cross | | | | |  |
| Sex | 1 | 0.02009631 | 1.40 | 0.238 | 0.00938 |
| Background | 7 | 0.010275356 | 0.717 | 0.658 | 0.0328 |
| cSOD Activity | 1 | 0.16066292 | 11.2 | **0.001** | 0.0704 |
| Sex X Background | 7 | 0.002961606 | 0.207 | 0.984 | 0.00967 |
| Background X cSOD Activity | 7 | 0.010056117 | 0.701 | 0.671 | 0.0321 |
| Sex X cSOD Activity | 1 | 0.00034346 | 0.0240 | 0.877 | 0.000162 |
| Sex X Background X cSOD Activity | 7 | 0.005488663 | 0.383 | 0.911 | 0.0178 |
| Error | 148 | 0.014339 |  |  |  |

Raw viability ANOVA outputs indicating significance of factors and their interactions, as well as the effect sizes of individual factors and interactions. Analysis of variance was used to test the factor effects with an alpha of 0.05. Effect sizes were calculated as partial eta squared (η_p_^2^). Bolded values denote significant factors in viability determination.

Table S3: Negative geotaxis ANOVA

| Source | d.f. | MS | F | P | η_p_^2^ |
| --- | --- | --- | --- | --- | --- |
| T0 - 0% and 50% cSOD Activity Cross | | | | |  |
| Sex | 1 | 0.198889 | 4.83 | **0.0294** | 0.0301 |
| Background | 7 | 0.103298 | 2.51 | **0.0179** | 0.101 |
| cSOD Activity | 1 | 6.977126 | 170 | **<.0001** | 0.521 |
| Sex X Background | 7 | 0.184849 | 4.49 | **0.0001** | 0.168 |
| Background X cSOD Activity | 7 | 0.231956 | 5.64 | **<.0001** | 0.202 |
| Sex X cSOD Activity | 1 | 0.094502 | 2.30 | 0.132 | 0.0145 |
| Sex X Background X cSOD Activity | 7 | 0.203628 | 4.95 | **<.0001** | 0.182 |
| Error | 156 | 0.041138 |  |  |  |
|  |  |  |  |  |  |
| T5 - 30% and 80% cSOD Activity Cross | | | | |  |
| Sex | 1 | 0.434153 | 8.80 | **0.0038** | 0.0809 |
| Background | 7 | 0.082013 | 1.66 | 0.127 | 0.104 |
| cSOD Activity | 1 | 0.361305 | 7.33 | **0.008** | 0.0683 |
| Sex X Background | 7 | 0.14886 | 3.02 | **0.0064** | 0.174 |
| Background X cSOD Activity | 7 | 0.07907 | 1.60 | 0.143 | 0.101 |
| Sex X cSOD Activity | 1 | 0.088575 | 1.80 | 0.183 | 0.0176 |
| Sex X Background X cSOD Activity | 7 | 0.100123 | 2.03 | 0.0585 | 0.124 |
| Error | 100 | 0.049318 |  |  |  |

Raw negative geotaxis ANOVA outputs indicating significance of factors and their interactions, as well as the effect sizes of individual factors and interactions. Analysis of variance was used to test the factor effects with an alpha of 0.05. Effect sizes were calculated as partial eta squared (η_p_^2^). Bolded values denote significant factors in negative geotaxis determination.

Table S4: Countercurrent ANOVA

| Source | d.f. | MS | F | P | η_p_^2^ |
| --- | --- | --- | --- | --- | --- |
| T0 - 0% and 50% cSOD Activity Cross | | | | |  |
| Sex | 1 | 0.204442 | 3.55 | 0.0606 | 0.0130 |
| Background | 7 | 0.045908 | 0.797 | 0.590 | 0.0203 |
| cSOD Activity | 1 | 28.84678 | 501 | **<.0001** | 0.651 |
| Sex X Background | 7 | 0.025527 | 0.443 | 0.874 | 0.0114 |
| Background X cSOD Activity | 7 | 0.026691 | 0.464 | 0.860 | 0.0119 |
| Sex X cSOD Activity | 1 | 0.000381 | 0.00660 | 0.935 | 2.46E-05 |
| Sex X Background X cSOD Activity | 7 | 0.038725 | 0.673 | 0.695 | 0.0172 |
| Error | 269 | 0.05758 |  |  |  |
|  |  |  |  |  |  |
| T5 - 30% and 80% cSOD Activity Cross | | | | |  |
| Sex | 1 | 1.596702 | 20.8 | **<.0001** | 0.106 |
| Background | 7 | 0.069567 | 0.906 | 0.503 | 0.0350 |
| cSOD Activity | 1 | 4.728976 | 61.6 | **<.0001** | 0.260 |
| Sex X Background | 7 | 0.123302 | 1.61 | 0.136 | 0.0604 |
| Background X cSOD Activity | 7 | 0.028009 | 0.365 | 0.922 | 0.0144 |
| Sex X cSOD Activity | 1 | 0.274622 | 3.58 | 0.0602 | 0.0200 |
| Sex X Background X cSOD Activity | 7 | 0.128282 | 1.67 | 0.119 | 0.0627 |
| Error | 175 | 0.076765 |  |  |  |

Raw countercurrent ANOVA outputs indicating significance of factors and their interactions, as well as the effect sizes of individual factors and interactions. Analysis of variance was used to test the factor effects with an alpha of 0.05. Effect sizes were calculated as partial eta squared (η_p_^2^). Bolded values denote significant factors in climbing ability determination.

**Table S5: MEN A**ctivity ANOVA

| Source | d.f. | MS | F | P | η_p_^2^ |
| --- | --- | --- | --- | --- | --- |
| T0 - 0% and 50% cSOD Activity Cross | | | | | |
| Sex | 1 | 0.000433 | 27.2 | **<.0001** | 0.0929 |
| Background | 7 | 2.81E-05 | 1.76 | 0.0946 | 0.0445 |
| cSOD Activity | 1 | 3.62E-05 | 2.27 | 0.133 | 0.00850 |
| Sex X Background | 7 | 1.27E-05 | 0.795 | 0.592 | 0.0206 |
| Background X cSOD Activity | 7 | 1.9E-05 | 1.19 | 0.308 | 0.0305 |
| Sex X cSOD Activity | 1 | 0.000523 | 32.8 | **<.0001** | 0.110 |
| Sex X Background X cSOD Activity | 7 | 1.55E-05 | 0.973 | 0.452 | 0.0250 |
| Error | 265 | 0.000016 |  |  |  |
|  |  |  |  |  |  |
| T5 - 30% and 80% cSOD Activity Cross | | | | | |
| Sex | 1 | 0.001063 | 38.0 | **<.0001** | 0.119 |
| Background | 7 | 4.86E-05 | 1.74 | 0.101 | 0.0416 |
| cSOD Activity | 1 | 0.000112 | 4.01 | **0.0461** | 0.0141 |
| Sex X Background | 7 | 8.9E-05 | 3.18 | **0.003** | 0.0736 |
| Background X cSOD Activity | 7 | 8.85E-05 | 3.16 | **0.0031** | 0.0733 |
| Sex X cSOD Activity | 1 | 0.000295 | 10.5 | **0.0013** | 0.0363 |
| Sex X Background X cSOD Activity | 7 | 9.68E-06 | 0.346 | 0.932 | 0.00857 |
| Error | 280 | 0.000028 |  |  |  |

Raw MEN activity ANOVA outputs indicating significance of factors and their interactions, as well as the effect sizes of individual factors and interactions. Analysis of variance was used to test the factor effects with an alpha of 0.05. Effect sizes were calculated as partial eta squared (η_p_^2^). Bolded values denote significant factors in MEN activity determination.

**Table S6: IDH A**ctivity ANOVA

| Source | d.f. | MS | F | P | η_p_^2^ |
| --- | --- | --- | --- | --- | --- |
| T0 - 0% and 50% cSOD Activity Cross | | | | | |
| Sex | 1 | 9.3E-07 | 0.271 | 0.603 | 0.00102 |
| Background | 7 | 4.98E-06 | 1.44 | 0.188 | 0.0368 |
| cSOD Activity | 1 | 0.000193 | 56.0 | **<.0001** | 0.174 |
| Sex X Background | 7 | 4.06E-06 | 1.18 | 0.315 | 0.0302 |
| Background X cSOD Activity | 7 | 4.49E-06 | 1.30 | 0.250 | 0.0332 |
| Sex X cSOD Activity | 1 | 8.81E-05 | 25.5 | **<.0001** | 0.0879 |
| Sex X Background X cSOD Activity | 7 | 2.97E-06 | 0.861 | 0.538 | 0.0222 |
| Error | 265 | 3.45E-06 |  |  |  |
|  |  |  |  |  |  |
| T5 - 30% and 80% cSOD Activity Cross | | | | | |
| Sex | 1 | 3E-05 | 4.58 | **0.0333** | 0.0161 |
| Background | 7 | 2.51E-05 | 3.83 | **0.0005** | 0.0875 |
| cSOD Activity | 1 | 7.2E-07 | 0.109 | 0.741 | 0.000393 |
| Sex X Background | 7 | 1.51E-05 | 2.31 | **0.0263** | 0.0547 |
| Background X cSOD Activity | 7 | 1.14E-05 | 1.75 | 0.098 | 0.0419 |
| Sex X cSOD Activity | 1 | 4.1E-06 | 0.626 | 0.429 | 0.00223 |
| Sex X Background X cSOD Activity | 7 | 4.48E-06 | 0.685 | 0.685 | 0.0168 |
| Error | 280 | 6.55E-06 |  |  |  |

Raw IDH activity ANOVA outputs indicating significance of factors and their interactions, as well as the effect sizes of individual factors and interactions. Analysis of variance was used to test the factor effects with an alpha of 0.05. Effect sizes were calculated as partial eta squared (η_p_^2^). Bolded values denote significant factors in IDH activity determination.

**Table S7: G6PD A**ctivity ANOVA

| Source | d.f. | MS | F | P | η_p_^2^ |
| --- | --- | --- | --- | --- | --- |
| T0 - 0% and 50% cSOD Activity Cross | | | | | |
| Sex | 1 | 2.03E-05 | 22.0 | **<.0001** | 0.0766 |
| Background | 7 | 8.63E-06 | 9.36 | **<.0001** | 0.199 |
| cSOD Activity | 1 | 8.17E-05 | 88.6 | **<.0001** | 0.250 |
| Sex X Background | 7 | 2.44E-06 | 2.65 | **0.0117** | 0.0653 |
| Background X cSOD Activity | 7 | 1.39E-06 | 1.51 | 0.164 | 0.0383 |
| Sex X cSOD Activity | 1 | 1.81E-05 | 19.6 | **<.0001** | 0.0690 |
| Sex X Background X cSOD Activity | 7 | 2.37E-06 | 2.57 | **0.0142** | 0.0635 |
| Error | 265 | 9.23E-07 |  |  |  |
|  |  |  |  |  |  |
| T5 - 30% and 80% cSOD Activity Cross | | | | | |
| Sex | 1 | 0.00037 | 155 | **<.0001** | 0.357 |
| Background | 7 | 1E-05 | 4.20 | **0.0002** | 0.0951 |
| cSOD Activity | 1 | 4.7E-06 | 1.97 | 0.161 | 0.00700 |
| Sex X Background | 7 | 3.97E-06 | 1.67 | 0.117 | 0.0400 |
| Background X cSOD Activity | 7 | 6.31E-06 | 2.65 | **0.0114** | 0.0621 |
| Sex X cSOD Activity | 1 | 9.4E-07 | 0.394 | 0.531 | 0.00141 |
| Sex X Background X cSOD Activity | 7 | 3.87E-06 | 1.62 | 0.128 | 0.0390 |
| Error | 280 | 2.38E-06 |  |  |  |

Raw G6PD activity ANOVA outputs indicating significance of factors and their interactions, as well as the effect sizes of individual factors and interactions. Analysis of variance was used to test the factor effects with an alpha of 0.05. Effect sizes were calculated as partial eta squared (η_p_^2^). Bolded values denote significant factors in G6PD activity determination.

**Table S8: Summary of Maximum and Minimum Line Effects**

| cSOD |  |  | Longevity | Viability | Negative | Climbing | MEN | IDH | G6PD |
| --- | --- | --- | --- | --- | --- | --- | --- | --- | --- |
| Activity | Sex | Status |  |  | Geotaxis | Ability | Activity | Activity | Activity |
| 0% | Male | High Line | **313** | 335 | **307** | 517 | 517 | 820 | **313** |
|  | Male | Low Line | **335** | 313 | **705** | 313 | 307 | 705 | **307** |
|  | Female | High Line | **820** | 324 | **313** | 820 | 307 | 820 | **517** |
|  | Female | Low Line | **304** | 705 | **517** | 517 | 313 | 705 | **324** |
| 50% | Male | High Line | **705** | 705 | **517** | 307 | 705 | 517 | **517** |
|  | Male | Low Line | **307** | 304 | **307** | 705 | 304 | 304 | **307** |
|  | Female | High Line | **820** | 705 | **517** | 307 | 705 | 324 | **324** |
|  | Female | Low Line | **517** | 335 | **304** | 313 | 517 | 335 | **307** |
| 30% | Male | High Line | **705** | 517 | **307** | 335 | **517** | **304** | **517** |
|  | Male | Low Line | **304** | 304 | **335** | 304 | **705** | **705** | **307** |
|  | Female | High Line | **335** | 517 | **307** | 517 | **304** | **304** | **304** |
|  | Female | Low Line | **304** | 324 | **304** | 335 | **324** | **705** | **324** |
| 80% | Male | High Line | **705** | 820 | **820** | 335 | **307** | **517** | **517** |
|  | Male | Low Line | **820** | 335 | **335** | 324 | **335** | **313** | **335** |
|  | Female | High Line | **324** | 517 | **307** | 307 | **307** | **304** | **705** |
|  | Female | Low Line | **820** | 335 | **820** | 324 | **324** | **313** | **324** |

Summary of maximum and minimum line effects for longevity, viability, negative geotaxis, countercurrent (climbing ability), malic enzyme (MEN), isocitrate dehydrogenase (IDH), and glucose-6-phosphate dehydrogenase (G6PD). Analysis of variance was used to test the factor effects with an alpha of 0.05. Effect sizes were calculated as partial eta squared (η_p_^2^). Bolded lines reflect phenotypes that demonstrated significant line effects**.**

**Table S9: Summary of the Average Genotype Values**

|  |  | cSOD | Longevity | Viability | Negative | Climbing | MEN | IDH | G6PD |
| --- | --- | --- | --- | --- | --- | --- | --- | --- | --- |
| cSOD |  | Activity | (Days) | (Frequency) | Geotaxis | Ability | Activity | Activity | Activity |
| Activity | Sex | Units/ug |  |  | (PI) | (CF) | Units/ug | Units/ug | Units/ug |
| 0% | Male | 0.000192±0.000048 | 5.7±0.1 | 0.211±0.012 | 0.41±0.05 | 0.30±0.03 | 0.0231±0.0004 | 0.00706±0.00015 | 0.00501±0.00011 |
|  | Female | 0.000106±0.000016 | 8.5±0.2 | 0.195±0.014 | 0.38±0.05 | 0.23±0.03 | 0.0234±0.0006 | 0.00827±0.00024 | 0.00503±0.00012 |
| 50% | Male | 0.000875±0.00010 | 57.1±0.5 | 0.339±0.012 | 0.84±0.02 | 0.93±0.02 | 0.0252±0.0004 | 0.00974±0.00024 | 0.00659±0.00015 |
|  | Female | 0.000883±0.00012 | 66.6±0.8 | 0.330±0.012 | 0.73±0.03 | 0.88±0.03 | 0.0200±0.0004 | 0.00874±0.00022 | 0.00555±0.00011 |
| 30% | Male | 0.000408±0.000045 | 48.2±0.7 | 0.237±0.014 | 0.79±0.06 | 0.69±0.05 | 0.0278±0.0005 | **0.00976±0.00025** | **0.00846±0.00019** |
|  | Female | 0.000546±0.000069 | 55.9±0.8 | 0.261±0.017 | 0.63±0.06 | 0.47±0.05 | 0.0214±0.0007 | **0.00915±0.00034** | **0.00601±0.00017** |
| 80% | Male | 0.00184±0.00015 | 54.4±0.8 | 0.299±0.017 | 0.86±0.03 | 0.95±0.01 | 0.0245±0.0005 | **0.01017±0.00035** | **0.00810±0.00022** |
|  | Female | 0.00253±0.00021 | 61.7±1.1 | 0.318±0.020 | 0.79±0.03 | 0.85±0.04 | 0.0227±0.0007 | **0.00923±0.00027** | **0.00595±0.00016** |

Summary of genotype values, pooled across genetic background, for longevity, viability, negative geotaxis, countercurrent (climbing ability), malic enzyme (MEN), isocitrate dehydrogenase (IDH), and glucise-6-phosphate dehydrogenase (G6PD). Analysis of variance was used to test the factor effects with an alpha of 0.05. Effect sizes were calculated as partial eta squared (η_p_^2^). Bolded lines reflect phenotypes that did not demonstrate significant cSOD activity effects**.**

**Table S10: Maximum and Minimum Line Value S**ummaries

|  |  |  | Longevity | Viability | Negative | Climbing | MEN | IDH | G6PD |
| --- | --- | --- | --- | --- | --- | --- | --- | --- | --- |
| cSOD |  |  | (Days) | (Frequency) | Geotaxis | Ability | Activity | Activity | Activity |
| Activity | Sex | Status |  |  | (PI) | (CF) | Units/ug | Units/ug | Units/ug |
| 0% | Male | Low Line | **4.75±0.22** | 0.186±0.046 | **0** | 0.151±0.069 | 0.0218±0.0015 | 0.00600±0.00037 | **0.00393±0.00031** |
|  | Male | High Line | **8.38±0.53** | 0.245±0.025 | **0.778±0.062** | 0.391±0.11 | 0.0241±0.0011 | 0.00797±0.00062 | **0.00568±0.00048** |
|  | Female | Low Line | **6.58±0.38** | 0.126±0.029 | **0.175±0.094** | 0.153±0.059 | 0.0214±0.0017 | 0.00741±0.00043 | **0.00456±0.00047** |
|  | Female | High Line | **13.4±0.62** | 0.266±0.054 | **0.702±0.098** | 0.317±0.21 | 0.0250±0.0015 | 0.00896±0.00071 | **0.00551±0.00030** |
| 50% | Male | Low Line | **50.8±2.3** | 0.306±0.039 | **0.772±0.052** | 0.867±0.11 | 0.0213±0.00096 | 0.00835±0.00026 | **0.00518±0.00019** |
|  | Male | High Line | **64.5±1.2** | 0.389±0.025 | **0.905±0.030** | 0.990±0.010 | 0.0277±0.0016 | 0.0110±0.00073 | **0.00840±0.00043** |
|  | Female | Low Line | **58.7±1.8** | 0.293±0.015 | **0.507±0.074** | 0.798±0.097 | 0.0184±0.0011 | 0.00801±0.00045 | **0.00483±0.00039** |
|  | Female | High Line | **74.4±2.1** | 0.366±0.022 | **0.846±0.050** | 0.950±0.050 | 0.0225±0.00070 | 0.0104±0.00074 | **0.00637±0.00038** |
| 30% | Male | Low Line | **42.4±4.2** | 0.189±0.052 | **0.500±0.29** | 0.463±0.20 | **0.0252±0.0014** | **0.00830±0.00033** | **0.00758±0.00065** |
|  | Male | High Line | **54.8±1.4** | 0.307±0.044 | **1** | 0.902±0.077 | **0.0301±0.00072** | **0.0109±0.0010** | **0.00922±0.00036** |
|  | Female | Low Line | **41.7±3.3** | 0.207±0.029 | **0.125±0.12** | 0.206±0.15 | **0.0164±0.00069** | **0.00692±0.00042** | **0.00508±0.00038** |
|  | Female | High Line | **60.8±1.5** | 0.304±0.053 | **0.938±0.063** | 0.740±0.080 | **0.0269±0.0042** | **0.0115±0.0024** | **0.00728±0.0010** |
| 80% | Male | Low Line | **41.4±3.0** | 0.236±0.035 | **0.724±0.12** | 0.900±0.10 | **0.0215±0.0016** | **0.00822±0.00076** | **0.00662±0.00047** |
|  | Male | High Line | **62.4±2.1** | 0.337±0.060 | **0.957±0.043** | 1 | **0.0273±0.0016** | **0.0127±0.00092** | **0.00971±0.00060** |
|  | Female | Low Line | **55.9±4.0** | 0.272±0.047 | **0.698±0.026** | 0.667±0.23 | **0.0194±0.0022** | **0.00843±0.00045** | **0.00541±0.00036** |
|  | Female | High Line | **65.1±2.8** | 0.362±0.010 | **0.892±0.10** | 0.984±0.016 | **0.0249±0.0021** | **0.00993±0.00088** | **0.00724±0.00055** |

Summary of maximum and minimum line values for longevity, viability, negative geotaxis, countercurrent (climbing ability), malic enzyme (MEN), isocitrate dehydrogenase (IDH), and glucise-6-phosphate dehydrogenase (G6PD). Analysis of variance was used to test the factor effects with an alpha of 0.05. Effect sizes were calculated as partial eta squared (η_p_^2^). Bolded lines reflect phenotypes that demonstrated significant line effects**.**
